# Role of CsrA in stress responses and metabolism important for *Salmonella* virulence revealed by integrated transcriptomics

**DOI:** 10.1371/journal.pone.0211430

**Published:** 2019-01-25

**Authors:** Anastasia H. Potts, Yinping Guo, Brian M. M. Ahmer, Tony Romeo

**Affiliations:** 1 Department of Microbiology and Cell Science, Institute of Food and Agricultural Sciences, University of Florida, Gainesville, FL, United States of America; 2 Department of Microbial Infection and Immunity, The Ohio State University, Columbus, OH, United States of America; East Carolina University Brody School of Medicine, UNITED STATES

## Abstract

To cause infection, *Salmonella* must survive and replicate in host niches that present dramatically different environmental conditions. This requires a flexible metabolism and physiology, responsive to conditions of the local milieu. The sequence specific RNA binding protein CsrA serves as a global regulator that governs gene expression required for pathogenicity, metabolism, biofilm formation, and motility in response to nutritional conditions. Its activity is determined by two noncoding small RNAs (sRNA), CsrB and CsrC, which sequester and antagonize this protein. Here, we used ribosome profiling and RNA-seq analysis to comprehensively examine the effects of CsrA on mRNA occupancy with ribosomes, a measure of translation, transcript stability, and the steady state levels of transcripts under *in vitro* SPI-1 inducing conditions, to simulate growth in the intestinal lumen, and under in vitro SPI-2-inducing conditions, to simulate growth in the *Salmonella* containing vacuole (SCV) of the macrophage. Our findings uncovered new roles for CsrA in controlling the expression of structural and regulatory genes involved in stress responses, metabolism, and virulence systems required for infection. We observed substantial variation in the CsrA regulon under the two growth conditions. In addition, CsrB/C sRNA levels were greatly reduced under the simulated intracellular conditions and were responsive to nutritional factors that distinguish the intracellular and luminal environments. Altogether, our results reveal CsrA to be a flexible regulator, which is inferred to be intimately involved in maintaining the distinct gene expression patterns associated with growth in the intestine and the macrophage.

## Introduction

*Salmonella enterica* serovar Typhimurium (*Salmonella*) is a major cause of foodborne illness and diarrheal disease worldwide [1–7]. *Salmonella* utilizes a variety of dedicated virulence factors, many of which are encoded within horizontally acquired genomic regions called *Salmonella* pathogenicity islands (SPI)[8,9]. The best characterized of these are SPI-1 and SPI-2, each of which encodes a type III secretion system (T3SS)[10–12]. The SPI-1 T3SS is required for initial invasion of the intestinal epithelium, and its expression is controlled by the transcriptional regulators HilD and HilA [13–15]. A variety of other regulators that sense diverse environmental signals converge to control the expression of HilD and thus SPI-1 [16,17]. *In vitro* the SPI-1 T3SS is induced by late exponential growth in rich media [18,19]. The SPI-2 T3SS is important for later stages of infection, including establishment of a modified phagosomal compartment called the *Salmonella* containing vacuole (SCV) and manipulation of host cells to promote intracellular growth and survival [10,12,20–23]. The two-component regulatory system (TCS) SsrA-SsrB integrates a variety of regulatory signals to control SPI-2 expression [24–29]. SPI-2 expression is induced *in vitro* by growth in acidic minimal media with limiting concentrations of magnesium and phosphate or in late stationary phase in rich media [18]. There is significant crosstalk between regulators of the SPIs, and models with distinct roles for SPI-1 and SPI-2 in early and later stages of infection, respectively, are likely overly simplistic [18,24]. For example, HilD regulates expression of SPI-1 but it also controls SPI-2 expression in rich media by activating the transcription of the genes encoding its master regulator SsrA-SsrB [18,30].

CsrA is a post-transcriptional regulator that controls the expression of SPI-1 and SPI-2 through direct repression of *hilD* translation initiation during growth in rich media [31,32]. In addition, CsrA controls the expression of genes encoding regulators of metabolism and biofilm formation in *Salmonella* [33,34]. CsrA binds to conserved GGA motif(s) within its RNA targets, leading to changes in their structure, transcription elongation, translation initiation, and/or RNA stability [35]. It is the core component of the carbon storage regulatory (Csr) system that is highly conserved, especially in Gammaproteobacteria [35,36]. Across multiple species, CsrA tends to repress genes expressed in the stationary phase of growth and during exposure to stress [35,37]. In *E*. *coli* and *Salmonella*, the activity of CsrA is controlled primarily by the levels of two inhibitory small RNAs (sRNAs), CsrB and CsrC. These sRNAs contain many high affinity CsrA binding sites that sequester CsrA from its lower affinity mRNA targets [35]. The BarA-SirA (-UvrY) TCS is a critical regulator of CsrB/C expression [36]. This TCS is stimulated by the accumulation of short chain carboxylate compounds such as formate and acetate, which are abundant in the intestinal lumen and accumulate as *Salmonella* approaches stationary phase *in vitro* [38,39]. In *E*. *coli*, the alarmone (p)ppGpp that accumulates when cells undergo amino acid starvation or other nutritional stresses also stimulates the transcription of CsrB/C [36]. The presence of preferred carbon sources such as glucose stimulates CsrB/C decay through a pathway involving unphosphorylated EIIA^Glc^, CsrD, and RNase E [40]. Thus, end products of metabolism and amino acid starvation stimulate CsrB/C transcription, and the abundance of preferred carbon sources lead to CsrB/C turnover, at least in closely related *E*. *coli* [36,38,40]. Together, these factors fine-tune CsrB/C expression based on the metabolic state of the cell. Control of virulence gene expression by CsrA is just one example of how nutrition and metabolism are closely linked to virulence in *Salmonella* [41].

**Fig 1:**
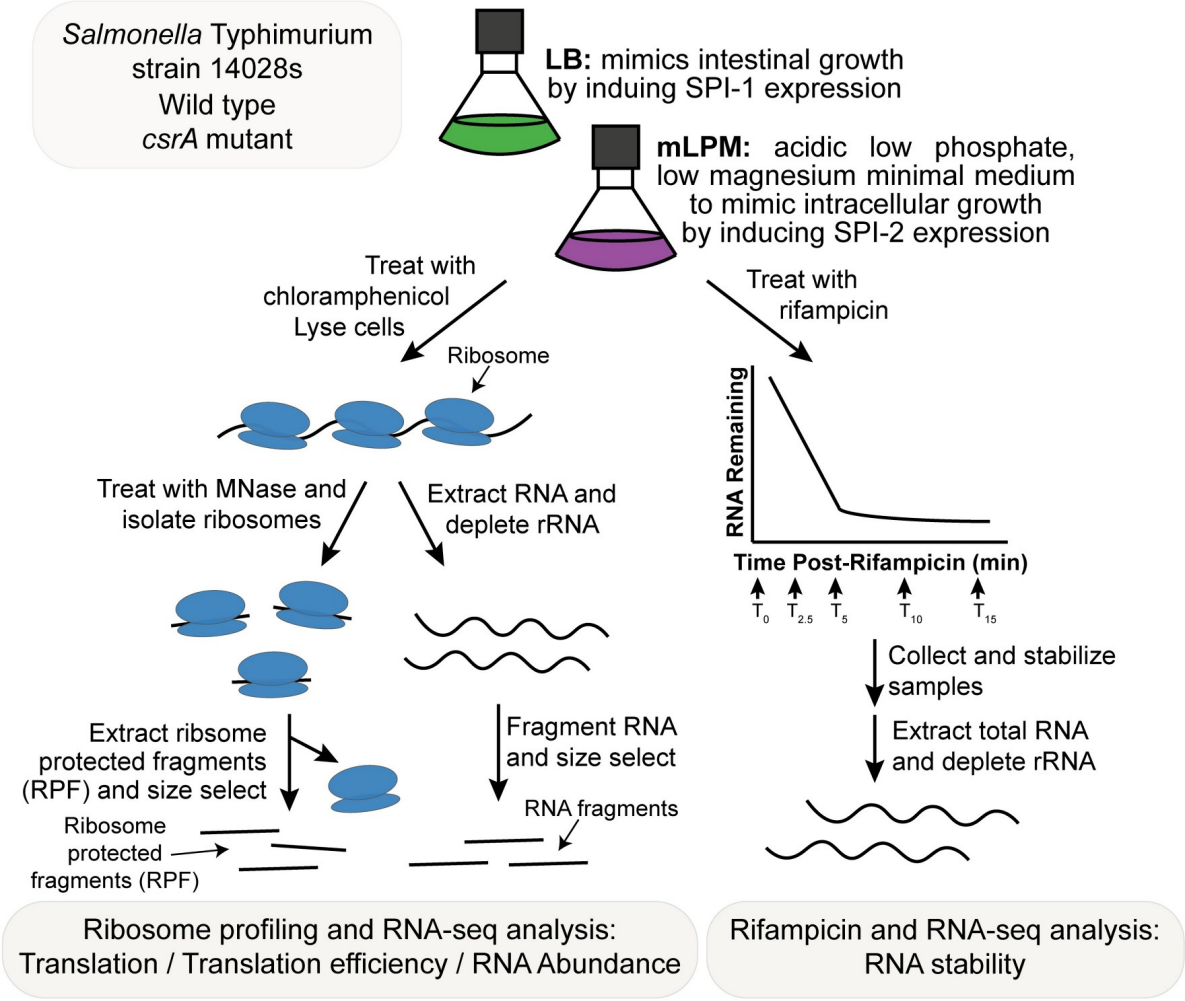
Overview of experimental design. Wild type *Salmonella enterica* serovar Typhimurium strain 14028s and an isogenic *csrA* mutant strain were grown in LB and mLPM media to mid-exponential phase. For analyses of translation, RNA abundance, and translation efficiency, cultures were treated with chloramphenicol to stall translation elongation. Samples were then quickly isolated and lysed under ribosome stabilizing conditions. Part of this lysate was treated with micrococcal nuclease, and ribosomes were isolated by ultracentrifugation through a sucrose cushion. Ribosome protected fragments (RPF) were extracted, size selected, and used to generate sequencing libraries reflecting the actively translated RNA. Another part of the lysate was used to extract total RNA. This RNA was depleted of rRNA, fragmented, and size selected. These RNA fragments were used to generate sequencing libraries reflecting steady state RNA levels. For analysis of RNA stability, cultures were treated with rifampicin to stop transcription initiation. Samples were collected at 0, 2.5, 5, 10, and 15 minutes after the addition of rifampicin and immediately stabilized with a phenol/ethanol stop solution. Total RNA was purified and depleted of rRNA. Sequencing libraries were prepared that reflect the abundance of RNA as it decays after the addition of rifampicin.

Studies have shown that deletion of *csrA* results in diminished *Salmonella* invasion of epithelial cells and virulence in mouse models [32,42]. Likely, CsrA control of HilD expression is an important mediator of this effect [31], but in other species CsrA regulates a variety of genes involved in stress responses and metabolism that may also contribute [35]. Here we aimed to further explore the regulatory role of CsrA under conditions relevant for infection. Specifically, we assessed the impact of CsrA on gene expression during growth in LB, a rich medium that induces SPI-1 expression [18], and mLPM, an acidic, low phosphate and magnesium minimal medium that favors SPI-2 expression [18,43]. We used ribosome profiling and RNA-seq to measure the effect of CsrA on translation, RNA abundance, and RNA stability in both of these conditions (Fig 1). A partial *csrA* disruption mutant strain encoding a truncated, functionally impaired CsrA protein [44] was used for these studies rather than a *csrA* deletion strain, which grows very slowly and is genetically unstable [32]. This CsrA allele expresses a protein that can still bind to RNA, though with an eight-fold lower affinity [44]. Unlike the deletion mutant, the CsrA truncation mutant grows normally in both LB and mLPM (S1 Fig). The integrated results of these analyses illustrate a global regulatory role of CsrA in *Salmonella* metabolism, virulence, and stress responses, which appears to contribute to its success as a pathogen by helping to maintain the distinct gene expression patterns needed for growth and survival in the intestine and the macrophage.

## Results

### CsrA is primarily a repressor of steady state RNA abundance, translation, and translation efficiency

Ribosome profiling is an RNA footprinting method that analyzes ribosomal occupancy of mRNA as a measure of apparent translation, at a genomic level by sequencing nuclease-resistant, ribosome-protected RNA fragments (RPF)[45](Fig 1). It has been used to study translation and post-transcriptional regulation in both prokaryotes and eukaryotes [45,46]. When combined with RNA-seq, ribosome profiling can be used to determine translation efficiency, a measure of post-transcriptional regulation. Here we defined translation efficiency as an interaction term between changes in RPF and RNA abundance, i.e. measuring changes in translation while controlling for changes in RNA abundance. As we expect that CsrA will indirectly affect the transcription of many genes, this will result in changes in RNA abundance and therefore translation of indirect targets. However, the translational efficiency of a gene would only be affected if there were a change in its post-transcriptional regulation. Although a change in translational efficiency indicates a changes in post-transcriptional regulation, interpreting this is not straightforward due to the intimiate link between transcription, translation, and RNA stability in bacteria. For example, repression of translation can alter the rate of mRNA decay. We used ribosome profiling and paired RNA-seq in an integrated analysis of differential translation (RPF levels), steady state RNA abundance (RNA levels), and translation efficiency (RPF and RNA levels) of protein coding genes in the wild type and *csrA* mutant strains grown in LB and mLPM media (Fig 1). In addition, we also separately analyzed changes in RNA abundance of protein- and non-protein coding genes. For each sample type, the four biological replicates form tight distinct clusters in a multidimensional scaling plot, suggesting that the approach is reproducible (S2 Fig). Our data replicated previous findings that growth in LB induces SPI-1 expression, whereas growth in mLPM induces SPI-2 expression [18,43] (S1 Table). In addition, CsrA generally acted as a repressor of translation, translation efficiency, and RNA abundance in both mLPM and LB (S2 Fig and S2 Table).

### CsrA is a global stabilizer of RNA

As CsrA directly regulates the stability of some of its RNA targets [35], we also assessed the effect of CsrA on RNA abundance after treatment with rifampicin to arrest transcription initiation (Fig 1). RNA-seq was used to measure RNA abundance, and normalized abundances were used to calculate RNA half-lives. The RNA-seq data were normalized with scaling factors calculated by comparing absolute RNA abundances measured with qRT-PCR to RNA-seq, as described previously [47]. A multidimensional scaling plot shows the reproducibility of half-lives calculated with this approach due to tight clustering of the biological replicates (S2 Fig). The mean RNA half-life and the individual half-life distribution were significantly different for the wild type vs. *csrA* mutant strains in both mLPM (2.6 vs. 1.5 min) and LB media (4.5 vs. 2.6 min), where CsrA acted in both cases to globally stabilize RNAs (Wilcox rank sum test, p < 0.0005, S4 Fig). This is in agreement with recent reports in *E*. *coli* [48,49].

### Integration of multiple transcriptomics analyses

**Fig 2:**
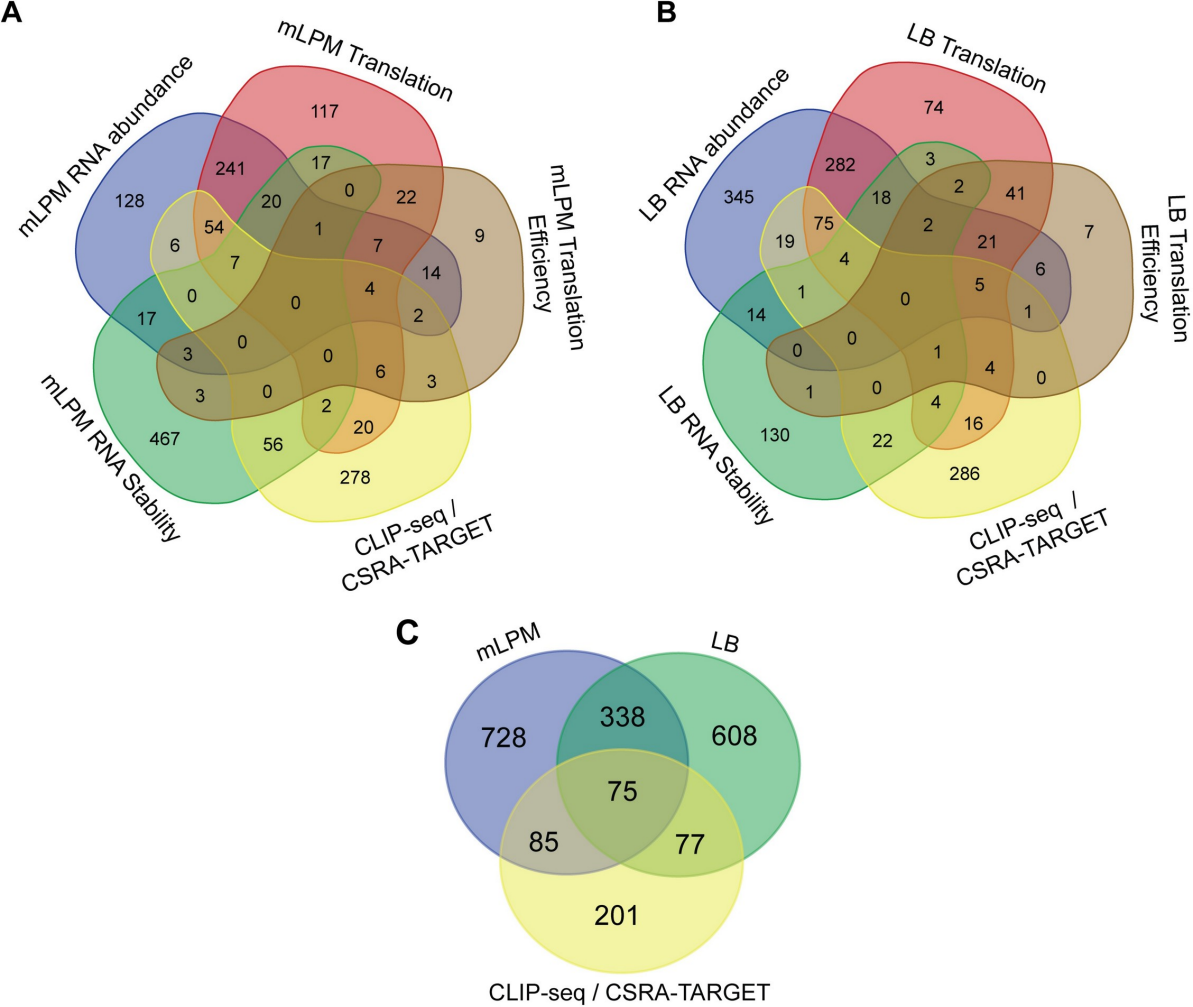
Integration of results from different transcriptomics methods. Number of genes with changes in their expression in (A) mLPM, (B) LB, and (C) a comparison between mLPM and LB. Data for predicted direct CsrA targets was taken from Kulkarni et al. [53] (CSRA-TARGET) and Homlqvist et al. [52] (CLIP-seq).

Coordinate changes in gene expression identified with multiple methods would provide strong evidence for CsrA-dependent regulation. Indeed, there was substantial overlap between the genes identified with multiple methods for both mLPM and LB (Fig 2). However, many genes with changes in their RNA stability did not have changes in their RNA abundance. A potential cause of this finding may be that there are compensatory effects on the transcription of these genes, as described previously for CsrB/C and DksA [50,51].

To help interpret the changes in gene expression, we also made use of the results from previous studies aimed at identifying direct binding targets of CsrA [52,53]. A recent study used CLIP-seq to identify CsrA RNA binding partners in *Salmonella* Typhimurium strain SL1344 during stationary phase growth in LB media [52]. Another study used the consensus CsrA binding site determined using systematic evolution of ligands by exponential enrichment (SELEX) to identify potential CsrA binding sites *in silico* in the *Salmonella* Typhimurium strain LT2 genome [53]. We used these data to enrich our interpretation of changes in gene expression (S3 Table). However, as the CLIP-seq study was conducted under different growth conditions it may not identify genes relevant under our conditions and vice versa. In addition, few of the *in silico* predicted CsrA targets have been verified *in vitro* or *in vivo*. An additional caveat is that as transcription, translation, and RNA stability are often intimately linked in bacteria [54], translational efficiency may be limited in its sensitivity. Thus, when a gene is associated with a signal in the CLIP-seq analysis, which we refer to as a CLIP-seq peak, and changes in translation but not with changes in translation efficiency, we still consider this to be suggestive of post-transcriptional regulation. Similar analyses conducted in *E*. *coli* also suggest that this is a reasonable hypothesis [48].

### Condition-specific effects of CsrA

Examination of the overlap between genes regulated by CsrA in mLPM and LB indicated that CsrA regulated many genes under only one condition (Fig 2C). Closer analysis of the log_2_ transformed fold change effects of CsrA in mLPM versus LB and their associated Spearman’s correlations highlighted the condition specific effects for translation (ρ = 0.34), RNA abundance (ρ = 0.20), translation efficiency (ρ = 0.11), and RNA stability (ρ = 0.23) (S5 Fig). These correlation coefficients suggested there is not a strong relationship between the effects of CsrA in these two conditions. We also formally analyzed these changes in gene expression by testing the statistical significance of the interaction between strain and media for ribosome profiling and RNA abundance and found that many genes were differentially regulated by CsrA between the two media (S4 Table). Together, these results suggest that there are strong condition-specific effects of CsrA on gene expression.

**Fig 3:**
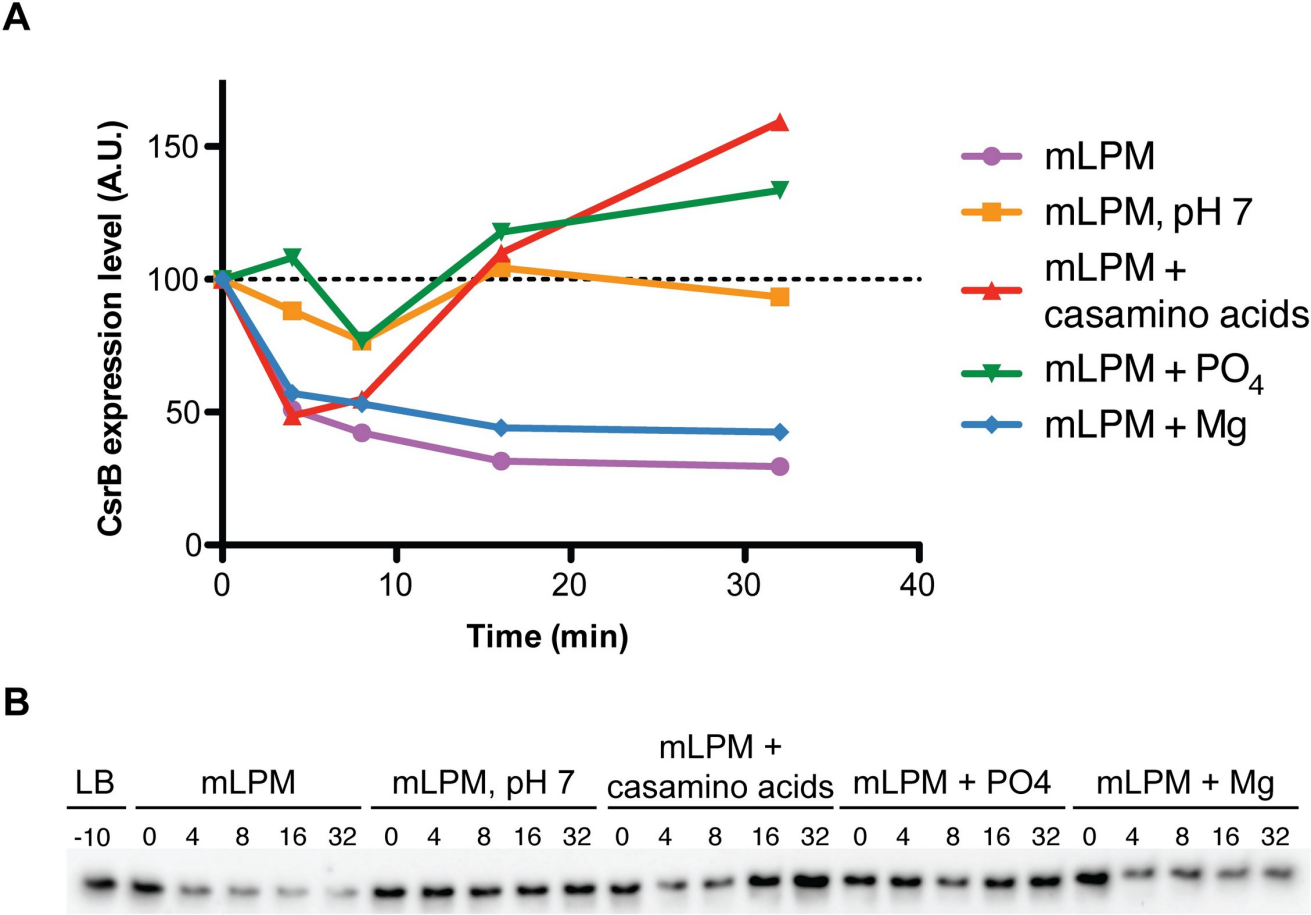
Exposure to mLPM medium reduces CsrB levels. (A) Levels of CsrB were analyzed during mid-exponential growth in LB medium and at several time points after transfer to several variations of mLPM medium. CsrB levels were quantified from the northern blot in (B) and displayed as arbitrary units (A.U.) after normalized to the 16s rRNA levels. Data from an additional experiment are presented in S6 Fig, showing that this response is reproducible.

There may be multiple explanations for the condition-specific effects. Transcription from alternative promoters under the different growth conditions may present alternative sites for CsrA-dependent regulation. CsrA regulation may also require coregulators that are expressed in only one condition. Conditionally regulated genes might be indirectly controlled by CsrA, leading to different effects on expression depending on condition specific cues or expression of regulators that are required to mediate CsrA effects. In fact, predicted and experimentally determined direct CsrA targets were significantly enriched (1.7 fold, two-sided Chi-square test, p < 0.0001) in genes that are expressed in both conditions (Fig 2C). This supports the idea that indirect regulation by CsrA is a major contributor to condition-specific effects. Finally, there may be differences in the expression or activity of Csr system components between LB and mLPM media. There was no apparent change in CsrA, BarA, or SirA expression, but CsrB and CsrC levels were substantially decreased, 8.1- and 3.3-fold, respectively, in mLPM relative to LB (S1 Table). This is consistent with the previously observed decrease in CsrB/C levels during intracellular growth in macrophages versus growth in LB [55]. We confirmed that exposure to mLPM leads to a sharp decrease in CsrB levels relative to mid-exponential growth in LB (Fig 3A and 3B and S6 Fig). In addition, the acidic pH, carbon source, and the low level of phosphate in mLPM contributed to this change in CsrB levels (Fig 3A and 3B and S6 Fig). This is consistent with a large transcriptomic study that found that mild acid shock leads to a decrease in CsrB and CsrC levels in LB medium [56]. We would predict that such a sharp decrease in the levels of CsrB/C should greatly increase the activity of CsrA in mLPM compared to LB. However, overall we did not observe a global increase in the magnitude of CsrA-dependent effect on gene expression in mLPM (S5 Fig and S2 Table). This suggests that differences in CsrA-dependent regulation between the two media cannot be explained due to altered CsrB/C levels alone, and that there may be other regulators of CsrA in mLPM.

### Core and condition-specific roles of CsrA identified by functional enrichment

**Fig 4:**
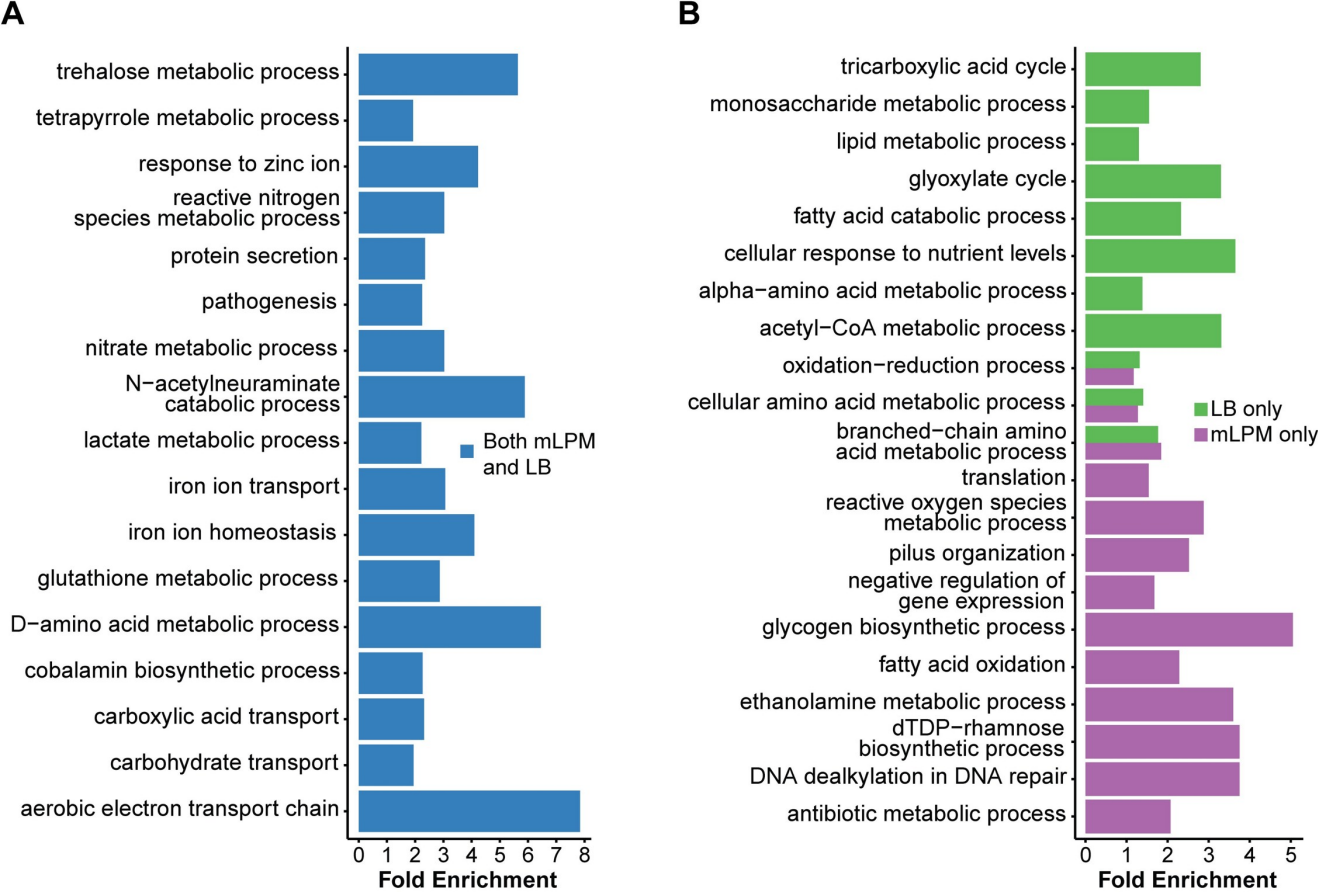
GO terms significantly enriched in CsrA regulated genes. Fold enrichment of GO terms in genes regulated by CsrA in both mLPM and LB (A) and in either LB or mLPM (B). Significance was determined with a Fisher’s exact test with TopGO [152]. Categories shown twice in panel B are indicative of larger lists of non-overlapping genes in mLPM and/or LB vs. genes in common. The list of significant terms (S6 Table) was shortened for visualization with REViGO [153].

To identify the functional roles of genes regulated by CsrA, we used enrichment analysis with Gene Ontology (GO) term annotations. Due to the strong condition specific effects, we analyzed genes regulated by CsrA in both mLPM and LB, those just in mLPM, and those just in LB (S6 Table and summarized in Fig 4). Several enriched functional categories include known regulatory roles of CsrA in *Salmonella*, such as in pathogenesis, type III secretion, and the use of ethanolamine as an electron donor [32,34,57](Fig 4). In addition, several functional roles of CsrA described in *E*. *coli* were also identified, such as regulation of the glycogen metabolism, the tricarboxylic acid cycle, and fatty acid metabolism [48,58–60]. Although the genes regulated by CsrA differed between mLPM and LB, there were some shared functional roles, such as amino acid metabolism (Fig 4B). On the other hand, several functions were only enriched in one condition, including the tricarboxylic acid cycle in LB and pilus expression in mLPM (Fig 4B). Several of these enriched GO terms are relevant to *Salmonella* infection, some of which will be explored in more detail in the following sections.

### CsrA regulates genes encoded in *Salmonella* pathogenicity islands and their regulators

Initial studies using a *csrA* deletion strain with unmapped suppressor mutation(s) found that both deletion and overexpression of *csrA* led to decreased HilD and SPI-1 expression in *Salmonella* [32,34]. On the other hand, the BarA-SirA TCS has been shown to activate SPI-1 and SPI-2 expression through its activation of CsrB/C, which relieves direct CsrA-mediated repression of HilD [31,36]. The later study demonstrating that CsrA directly represses HilD translational initiation illustrates the only known molecular mechanism by which CsrA affects SPI-1 expression. We found that expression of SPI-1 encoded genes increased in the *csrA* disruption mutant in both LB and mLPM, with less pronounced effects on SPI-2 gene expression (S2 Table), which is consistent with the previous mechanistic results. In addition, we also observed that CsrA decreased the translational efficiency of HilD in LB and mLPM (S2 Table). It is unclear why our data do not agree with the initial studies showing that deletion of *csrA* decreases SPI-1 expression. Perhaps this reflects a difference between reduced CsrA activity in our mutant vs. elimination of all CsrA activity by the deletion. But growth differences genetic instability of the *csrA* deletion, or effects of the unknown suppressor(s) of this defect might also account for the difference.

**Fig 5:**
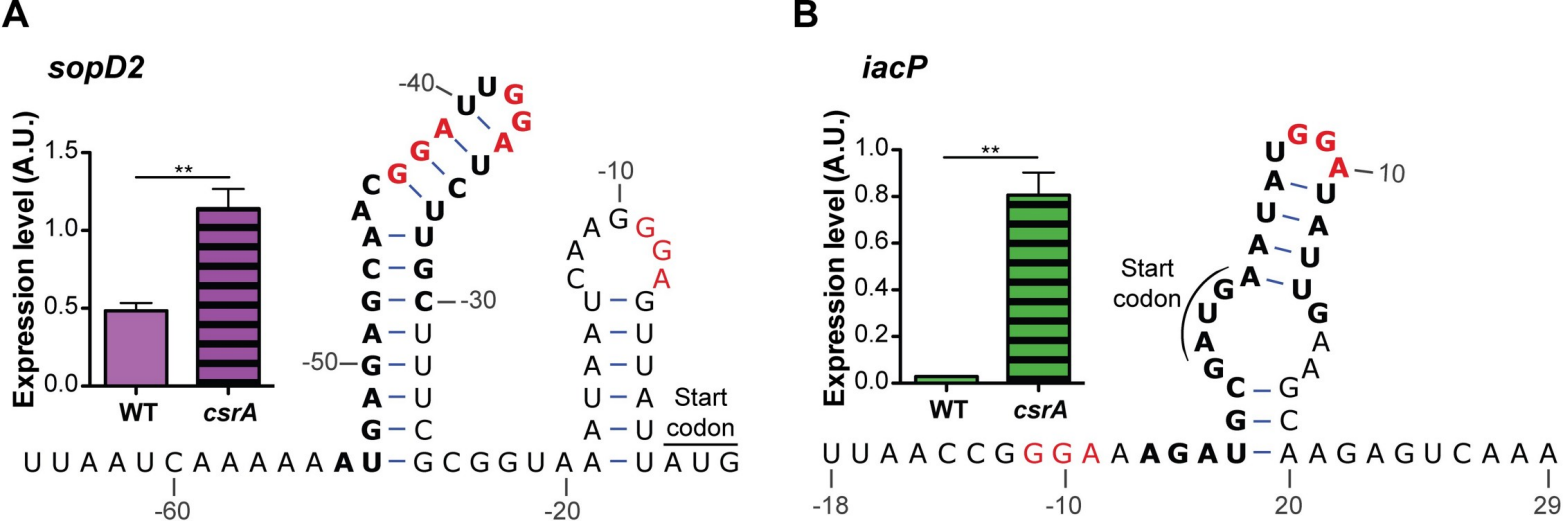
CsrA regulates genes encoded in *Salmonella* pathogenicity islands. RNA sequence and mFold [154] predicted structure of 5’-untranslated and early coding regions and qRT-PCR analysis of steady state mRNA levels of (A) *sopD2* and (B) *iacP*. GGA motifs are shown in red and CLIP-seq peaks from Holmqvist et al. [52] are shown in bold. RNA levels were assessed at mid-exponential growth in LB (green) or mLPM (purple) and normalized to the 16S rRNA levels. Error bars show SEM of three biological replicates. Statistical significance was determined with a two-sided student’s t-test. Asterisks denote statistical significance (p<0.05: *, p<0.005: **, p<0.0005: ***).

Recent studies have indicated that CsrA interacts *in vivo* with several mRNAs that encode T3SS effectors and genes encoded by SPI-1 and SPI-2 [52]. SopD2 is a SPI-2 secreted effector that is involved in disrupting host regulation of endocytic trafficking and promotes SCV stability [61,62]. Holmqvist et al. identified a CsrA CLIP-seq peak upstream of *sopd2* and found a *sopD2*’-‘*gfp* translation fusion was sensitive to changes in CsrB and CsrC expression [52]. Furthermore, mutation of the GGA motif within an apparent CsrA binding site in the 5’-UTR of the *sopD2*’-‘*gfp* fusion to CCU eliminated the effects of CsrB/C on its expression [52]. Consistent with these findings, we found that CsrA repressed the RNA abundance and translation of *sopD2* at its native locus in mLPM and LB (S2 Table), and we confirmed the effect on *sopD2* mRNA abundance with qRT-PCR (Fig 5A). Closer analysis of the 5’-UTR also showed a possible lower affinity CsrA binding site, containing a GGA motif at -9 to -7 relative to the translation initiation codon and overlapping the Shine Dalgarno sequence (SD) of *sopD2* (Fig 5A). This arrangement may allow a CsrA protein dimer to directly block 30S ribosome access to initiate translation by bridging from the upstream high affinity site to the SD, as shown previously for *glgC* mRNA [63].

CsrA also repressed the RNA abundance, translation, and translation efficiency of the SPI-1 encoded gene, *iacP* in LB (S2 Table). IacP is an acyl carrier protein that can acylate SPI-1 effectors, enhancing SopB, SopA, and SopD secretion and translocation [64,65]. An *iacP* mutant is attenuated for infection in mouse models of infection and exhibits decreased invasion of non-phagocytic cells [64]. A CsrA CLIP-seq peak was found overlapping the start of *iacP* [52](Fig 5B). A possible high affinity CsrA binding site lies within the CLIP-seq peak just within the coding region of *iacP*, and another GGA motif located within the SD sequence lies just upstream of the identified CLIP-seq peak in the 5’-UTR of *iacP* (Fig 5B). Plausibly, CsrA binding to these two GGA motifs blocks ribosome access to the SD. Indeed, the translation efficiency of *iacP* is enhanced in the *csrA* mutant (S2 Table). Although the level of *iacP* RNA was below the level of reliable detection, qRT-PCR was able to detect its expression and showed that CsrA represses accumulation of *iacP* mRNA (S2 Table, Fig 5B) in LB. These data suggest that CsrA has multiple inputs controlling genes located in SPI-1 (*hilD*, *iacP*) and genes of chromosomally encoded effectors secreted by the SPI-2 T3SS (*sopD2*). Such multi-layered regulation appears to be common for other complex phenotypes regulated by CsrA, such as biofilm formation [35,37].

In addition to its effects on T3SS effectors, CsrA controlled the expression of other regulators of *Salmonella* virulence. Predictably, CsrA repressed the expression of several transcriptional regulators under the control of HilD, which control SPI-1 and SPI-2 expression (*hilA*, *hilC*, *ssrA*, *ssrB*, *invF*, and *rtsA*) (S2 Table)[2,31]. CsrA repressed *spvR* translation 4-fold in mLPM, but levels of this mRNA were insufficient for analysis in LB (S2 Table). The virulence plasmid-encoded *spvR* gene is induced during growth in acidic minimal media and SpvR activates the expression of the SpvB and SpvC effector proteins [66,67]. These effectors aid non-typhoidal strains in mounting systemic infections and rely on the SPI-2 encoded T3SS for secretion. Consequently, *spvR* deletion strains are defective for virulence in mouse models of infection [42,66]. SlyA is a transcriptional regulator that coordinates with PhoP to induce the expression of SPI-2, thus contributing to intracellular survival and destruction of M cells [68–70]. CsrA repressed *slyA* translation 1.8-fold in LB but not in mLPM (S2 Table). SlyA is induced by growth in macrophages and in the stationary phase of growth in an *rpoS*-independent manner [71]. SlyA activates *pagC* and *sseA* expression in a PhoP-dependent manner [68,72,73], and only in LB was their translation repressed by CsrA 3.5- and 4.0-fold, respectively (S2 Table). This suggests that regulation of *slyA* by CsrA affects the expression of its downstream regulatory targets. Thus, in addition to the effects of CsrA on *hilD* expression, CsrA controlled the expression of regulators of other aspects of *Salmonella* virulence including repressing the activator of plasmid-encoded effectors *spvR* in mLPM and repressing the regulator of SPI-2 encoded effectors, *slyA* in LB. These effects may also contribute to the defects in virulence of a *csrA* mutant [32,34,42].

### CsrA represses genes required for resistance to stresses encountered during infection

During the course of infection, *Salmonella* encounters a variety of environmental stressors that it must survive in order to mount a successful infection [1,2,74]. The systems used to sense and respond to *in vivo* environmental conditions are important for *Salmonella* survival and growth within the host. Our functional enrichment analysis revealed several stress response pathways as enriched in CsrA regulated genes (Fig 4). RpoS (σ^s^) is involved in the activation of many of the genes required for stress resistance in *Salmonella* and is expressed during both early and late stages of infection [75,76]. CsrA repressed translation of *rpoS* 2.4-fold in LB but had no effect in mLPM (S2 Table). Thus, in LB CsrA may mediate some of its effects on stress responses through the master regulator of stress resistance, RpoS.

Before reaching the intestine, *Salmonella* must survive exposure to the acidic gastric contents of the stomach, which triggers the acid tolerance response (ATR)[77–79]. In addition, during intracellular growth *Salmonella* resides within the SCV, an acidic endosomal compartment [77–80]. In LB, CsrA repressed *adiAC* (S2 Table), which maintains intracellular pH by decreasing the concentration of intracellular protons via decarboxylation and exchange of arginine/agmatine. Previous work has shown that exogenous induction of *adiAC* is sufficient to protect *Salmonella* strains deficient of regulators of the ATR from killing by acidic pH [81]. CsrA also repressed expression of *cysB*, which encodes a LysR-family transcriptional regulator of cysteine biosynthesis that in *E*. *coli* is critical for expression of *adiA* and participates in the acid shock response [82,83]. In mLPM, CsrA repressed translation of *yciG*, which encodes a protein of unknown function that contributes strongly to *Salmonella* survival under acidic conditions [84]. CsrA also repressed translation of another acid-inducible gene in mLPM, *cfa* (S2 Table), which encodes cyclopropane fatty acid synthase [84]. By promoting cyclopropanation of unsaturated moieties of membrane fatty acids, this enzyme makes them more resistant to oxidation or chemical damage [85]. This plays a major role in *E*. *coli* survival during acid shock, especially during growth in minimal media when the amino acid decarboxylase systems are not functional [86]. Thus, CsrA repressed systems for the ATR in both mLPM and LB, which may facilitate ATR inducibility under conditions that increase CsrB/C levels.

*Salmonella* experiences changes in solute concentration as it encounters different host compartments, and must respond appropriately to osmotic stress during the course of infection [87]. Low moisture and high solute concentrations are also important conditions for food preservation, and mechanisms used to survive these conditions are relevant for foodborne *Salmonella* outbreaks [88]. Upon exposure to high solute concentrations, *Salmonella* attempts to maintain its turgor pressure by adjusting its intracellular solute concentration by inducing K^+^ pumps and either the transport or biosynthesis of osmoprotectants such as proline, glycine betaine, and trehalose [89]. Upon continuing stress, *Salmonella* then adjusts its outer membrane protein composition by activating expression of the smaller pore size OmpC over OmpF [88]. CsrA repressed the translation of *proP* 2.7-fold in mLPM (S2 Table), which encodes a permease that imports L-proline and glycine betaine, which is required for long term survival of *Salmonella* in low moisture environments [90]. In addition, CsrA repressed the translation of *otsBA* 3.0- and 2.2-fold in mLPM and LB, respectively (S2 Table). These genes encode enzymes required for the biosynthesis of trehalose and allow enhanced growth of *E*. *coli* during osmotic stress and at low temperatures [91,92]. CsrA also repressed translation of *osmY* 5.1- and 2.3-fold in mLPM and LB, respectively, which gives *E*. *coli* enhanced resistance to osmotic stress in rich media [93] (S2 Table). CsrA activated expression of both *ompF* and *ompC*, which encode outer membrane porins (S2 Table), and Holmqvist et al. identified a CLIP-seq peak in the 5’-UTR of *ompC* [52]. Overall, CsrA repressed a variety of genes required for osmotic stress resistance in *Salmonella* in both mLPM and LB.

Control of free iron in the intestinal lumen and blood is an important strategy used by the host to limit growth of pathogens, which is sometimes referred to as nutritional immunity [94]. Indeed, supplementation of iron in the diet has been shown to increase the prevalence and virulence of *Salmonella* in the intestine [95,96]. Due to limiting iron levels in the intestine, *Salmonella* must outcompete the native microbiota for access to this iron [97]. To do so, *Salmonella* uses siderophores, including enterobactin and its glycosylated derivative, salmochelin [98]. *Salmonella* can also scavenge iron that has been chelated by siderophores produced by other organisms including ferrioxamines and ferrichrome [99]. Our transcriptomics data revealed that in mLPM CsrA activated genes involved in enterobactin biosynthesis (*entCEAB* and *entF*), the outer membrane receptor for enterobactin (*fepA*), some genes involved salmochelin biosynthesis and transport (*iroBCD*), and the outer membrane receptor for ferrioxamines (*foxA*) (S2 Table). In LB, CsrA activated other genes involved in iron transport, including those involved in ferrichrome and ferrioxamine transport (*fhuACDB*), and ferrous iron transport (*feoABC*) (S2 Table). One possible mediator of these effects is *fur*, the master regulator of iron homeostasis [100,101]. However, CsrA did not regulate the expression of *fur* in mLPM or LB (S2 Table). These results suggest that CsrA plays a role in modulating the ability of *Salmonella* to acquire iron in the intestine and within host cells.

In addition to its effects on genes involved in iron acquisition, CsrA repressed translation of genes encoding the iron storage proteins *ftnB* (13- and 5.6-fold), *dps* (5.1- and 2.6-fold), and *bfr* (2.7-fold and not significant) in mLPM and LB, respectively (S2 Table). A CsrA CLIP-seq peak was associated with *bfr* (S2 Table)[52]. Bfr is the major iron storage protein in *Salmonella*, while FtnB and Dps contribute very little to intracellular iron levels [102]. FtnB contributes to the repair of oxidatively damaged iron sulfur clusters, while Dps protects DNA from oxidative damage [102,103]. Mutants of *ftnB* or *dps* have diminished *Salmonella* virulence in a mouse model of infection, whereas mutation of *bfr* has little or no effect on virulence [102]. Thus, repression of *ftnB* and *dps* by CsrA may be more closely related to repression of the oxidative stress response than to iron storage.

**Fig 6:**
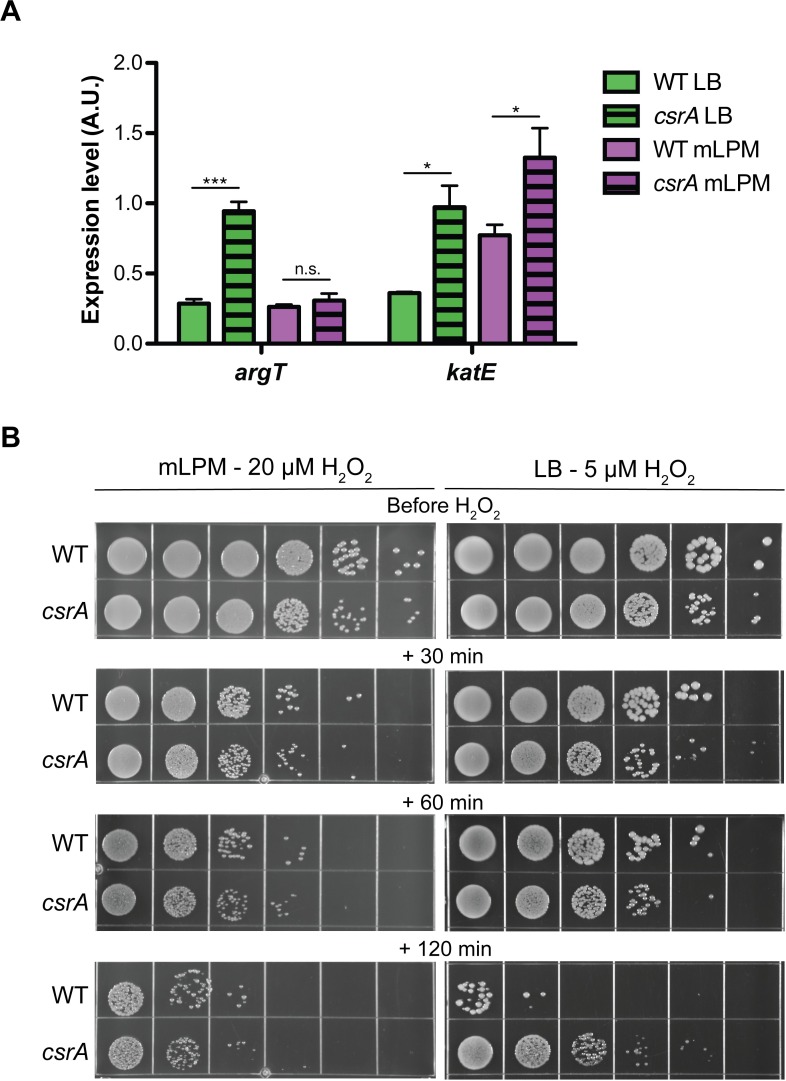
CsrA regulates oxidative stress responses. (A) qRT-PCR analysis of steady state *argT* and *katE* mRNA levels during mid-exponential growth in LB (green) or mLPM (purple) normalized to the 16S rRNA levels. Error bars show SEM of three biological replicates. Statistical significance was determined with a two-sided student’s t-test. Asterisks denote statistical significance (p<0.05: *, p<0.005: **, p<0.0005: ***). (B) Survival of wild type and *csrA* mutant strains upon exposure to H_2_O_2_. Strains in mid-exponential phase of growth in mLPM or LB were exposed to 20 μM or 5 μM of H_2_O_2_, respectively, for the indicated times. Washed and 10-fold serially diluted samples were plated and grown overnight on LB agar plates before imaging. Data from a biological replicate analyzed in the same experimented is presented in S7 Fig. Altogether, this experiment was repeated three times with similar results.

Other genes involved in oxidative stress resistance were also regulated by CsrA. It repressed translation of the catalases *katE* and *katN* 4.4- and 4.5-fold in mLPM only, respectively (S2 Table). Additional qRT-PCR experiments found that CsrA repressed accumulation of *katE* mRNA in both mLPM and LB (Fig 6A). CsrA also repressed translation of the superoxide dismutases *sodB* and *sodC2* in mLPM 2.2- and 2.5-fold, respectively (S2 Table). CsrA repressed translation of *btuE* 2-fold in mLPM (S2 Table), which encodes a peroxidase that can use thioredoxin or glutathione as a reductant [104]. CsrA also repressed genes involved in the glutaredoxin, thioredoxin, and glutathione systems in mLPM, including the glutaredoxin 3 (*grxC*), thioredoxin 2 (*trxC*), and three glutathione S-transferases (*yfcG*, *yqjG*, and STM14_5130) (S2 Table). These systems help maintain the redox status of the cell and protect against oxidative stress [105,106]. In LB, CsrA repressed the expression of the arginine permease *argT* (Fig 6A and S2 Table), which allows *Salmonella* to deplete arginine from its environment [107]. Arginine is a substrate for host nitric oxide synthases, so expression of *argT* helps *Salmonella* decrease the production of toxic nitric oxide [108]. In LB, CsrA also repressed expression of *slyA*, *rpoS*, and *soxS*, regulators that contribute to resistance to oxidative stress in *Salmonella* [71,109,110]. To determine if CsrA impacts the resistance to oxidative stress in *Salmonella*, we compared the susceptibility of wild type and *csrA* mutant strains to H_2_O_2_ in both LB and mLPM medium. Mutation of *csrA* resulted in a sharp increase in resistance to H_2_O_2_ in LB at 2 hr of exposure (Fig 6B), which is consistent with de-repression of genes involved in oxidative stress resistance. Growth in mLPM resulted in greater resistance to H_2_O_2_, and surprisingly, CsrA did not affect H_2_O_2_ sensitivity in mLPM (Fig 6B and S7 Fig). However, *Salmonella* encodes multiple redundant systems that can detoxify H_2_O_2_ and several must be deleted before a change in sensitivity is observed [111]. Often exposure to stresses can result in cross resistance to other stress. For example, pre-exposure to carbon limitation results in enhanced H_2_O_2_ resistance, which is mediated partly by RpoS. Thus, it is perhaps not surprising that the *Salmonella* is more resistant to oxidative stress in mLPM media, which is limiting for multiple critical nutrients. In contrast, the stress resistant phenotype may be apparent in LB because CsrA represses the expression of multiple regulators (*slyA*, *soxS*, and *rpoS*) that induce expression of genes required for oxidative stress resistance under this condtion.

### CsrA regulates metabolism important for establishing infection

One important contributor to success of *Salmonella* is its nutritional flexibility, including the ability to utilize many different carbon sources, electron donors, and electron acceptors [108]. CsrA regulated a variety of genes and regulators of alternative carbon source utilization (S2 Table). For example, in mLPM CsrA activated the translation of transcriptional regulators of genes involved in galactose (*galS*), rhamnose (*rhaSR*) and fucose (*fucR*) utilization (S2 Table). In LB, CsrA activated the translation of genes for fucose (*fucR*), 1,2-propanediol (*pocR*), and threonine/serine (*tdcA*) metabolism (S2 Table). With the exception of GalS, these regulators are all activators whose expression is required for induction of structural genes needed for catabolism of these nutrients. Thus, CsrA may have a role in the ability of *Salmonella* to sense and utilize these nutrients, of which galactose is important for growth in the SCV [55] while fucose and 1,2-propanediol are particularly important in the intestine [41].

**Fig 7:**
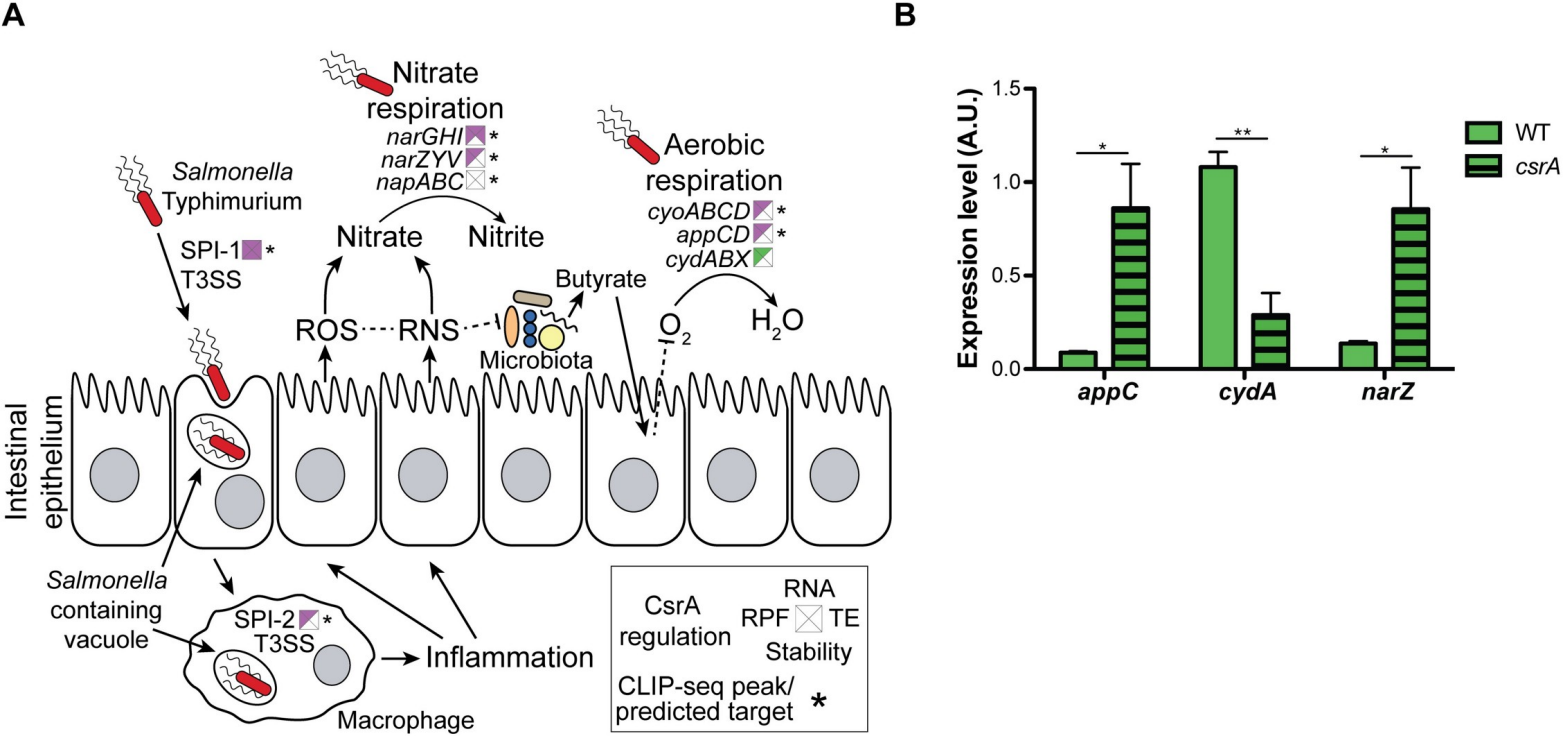
CsrA regulates metabolism important for establishing infection in the intestine. (A) Summary of the effect of CsrA on the expression of genes involved growth in the intestine. Squares represent the effect of CsrA on translation (RPF, left), RNA abundance (RNA, top), RNA stability (stability, bottom), and translational efficiency (TE, right). Purple indicates repression by CsrA, and green indicates activation. Asterisks show gene(s) that are associated with CLIP-seq peak(s) and/or were predicted to express CsrA-binding transcripts [52,53]. Genes of SPI-1 or SPI-2 are represented as a single entity although the specific genes vary in response to CsrA effects and as direct CsrA targets. S2 Table contains a full description of the observed effects. (B) Levels of steady state *appC* (*cyxA*), *cydA* and *narZ* mRNA levels during mid-exponential growth in LB normalized to the 16S rRNA levels measured with qRT-PCR and shown in arbitrary units (A.U.). Error bars show SEM of three biological replicates. Statistical significance was determined with a two-sided student’s t-test. Asterisks denote statistical significance (p<0.05: *, p<0.005: **, p<0.0005: ***).

CsrA modulates the expression of genes encoding terminal reductases for both aerobic and anaerobic respiration (Fig 7A and S2 Table). CsrA repressed translation of cytochrome oxidase bo (*cyoABCD*) an average of 2.3-fold in LB (S2 Table), and 3 CLIP-seq peaks were associated with this gene (S3 Table). In addition, CsrA repressed cytochrome oxidase bd-II (*appCD*) an average of 2.4- and 5.6-fold in LB and mLPM, respectively (S2 Table), confirmed with qRT-PCR in LB (Fig 7B). Cytochrome oxidase bo is expressed during aerobic growth when oxygen is abundant [112]. Recently, *appCD (cyxAB*) was found to contribute to post-antibiotic expansion of *Salmonella* and enhanced shedding in non-antibiotic treated mice [113]. After antibiotic treatment or *Salmonella* infection, depletion of butyrate producing *Clostridia* leads to an increase in the partial pressure of oxygen at the epithelium, which *Salmonella* exploits via *appCD*-mediated aerobic respiration [113]. On the other hand, CsrA activated translation of *cydABX* 3.5-fold in LB (S2 Table). Additional qRT-PCR experiments confirmed that CsrA activated *cydA* expression in LB (Fig 7B). This gene encodes cytochrome oxidase bd-I that plays an important role in low oxygen environments, such as the 5–10% oxygen found in tissues, due to its high affinity for oxygen [114]. Expression of cytochrome oxidase bd-I has also been shown to contribute significantly to the anti-nitrosative defenses of *Salmonella* during infection in addition to its contribution to respiration [114].

In addition to its effects on the expression of cytochrome oxidases, in LB CsrA regulated genes involved with anaerobic respiration relevant for *Salmonella* growth in the intestine (Fig 7A and S2 Table). CsrA repressed translation of nitrate reductase A (*narGHI*) and nitrate reductase Z (*narZYV*). The latter effects were supported by qRT-PCR examination of *narZ* transcript levels, and this gene was also identified as a predicted CsrA target (Fig 7B and S2 Table)[53]. Nitrate is produced in the intestine from the reaction of NO and O_2_^-^ produced by host enzymes as part of inflammation triggered by *Salmonella* infection [115]. CsrA also repressed expression of genes encoding formate dehydrogenase (*fdnGHI*) an average of 2.5-fold (S2 Table), which is active under anaerobic conditions and is involved in nitrate respiration [116]. High levels of formate in the intestine would provide ample substrate for this enzyme [39] and would decrease CsrA activity by inducing CsrB/C transcription [38,39]. CsrA also activated L-lactate dehydrogenase (*lldD*) 5.3-fold (S2 Table). L-lactate dehydrogenase contributes to aerobic metabolism, but it also can contribute to anaerobic nitrate respiration in *E*. *coli* [116,117]. Overall, our data suggest that CsrA has multiple regulatory inputs into expression of genes required for aerobic and anaerobic metabolism associated with its intestinal and intracellular niches.

## Discussion

To our knowledge, this is the first study to examine the global effects of CsrA on gene expression under more than one growth condition. As a result, we found that the growth condition is a strong determinant of CsrA-dependent regulation. There may be multiple explanations for the observed condition-specific effects. First, differences in CsrA activity between the two conditions may contribute. CsrA may also require coregulators expressed in only one condition. In addition, transcription from alternative promoters under the two conditions may present alternative sites for CsrA-dependent regulation. On the other hand, conditionally regulated genes may be indirectly responsive to CsrA via regulator(s) that are subject to the direct effects of CsrA as well as differentially affected by the two growth conditions. Regardless of the regulatory mechanisms, it is difficult to predict the effect of CsrA on gene expression under new growth conditions based on the wealth of data reported here. Nevertheless, our findings reveal that CsrA acts as a truly flexible global regulator of gene expression.

Our data also add to the increasing evidence that CsrA globally stabilizes RNA, while it paradoxically globally represses RNA abundance in both *E*. *coli* and *Salmonella* (Fig 2)[49]. On the other hand, few examples of RNA stabilization by CsrA have been studied in detail [35]. One recent study proposed that the overall stabilizing effect of CsrA was a consequence of its role as a repressor of RNA abundance because low abundance transcripts are fundamentally more stable than high abundance transcripts [49,118]. However, the present study and another in *E*. *coli* have shown that CsrA stabilizes many transcripts without significantly altering their abundance (S2 Table)[49]. In addition, this model relies on the hypothesis that enhanced RNA stability is a fundamental attribute of low RNA abundance due to a decreased probability of interaction with nucleases [118]. However, other studies in *E*. *coli* have concluded that mRNA stability is not predictive of abundance [119] and findings in other organisms have supported the opposite conclusion, that highly abundant RNAs are often more stable [47]. Existing models of the determinants of mRNA stability hold that differences in RNA stability are primarily a consequence of changes in the accessibility of nuclease cleavage sites and the expression of RNases [120]. Thus, the model that CsrA stabilizes transcripts as a consequence of its repression of RNA abundance is not clearly supported by existing evidence. CsrA may have an underappreciated global role in stabilizing RNA via direct RNA binding or may have indirect effect(s) via direct or indirect roles in the expression of other RNA binding proteins, nucleases or factors that globally influence RNA turnover. We did not find changes in expression of any RNases or components of the degradosome, which are responsible for bulk RNA turnover (S2 Table). CsrA in *E*. *coli* represses *hfq* and *pnp* expression [121,122], but we did not find effects on the expression of these genes in *Salmonella* (S2 Table). Without question, the role of CsrA in bulk RNA stabilization is worthy of further exploration.

**Fig 8:**
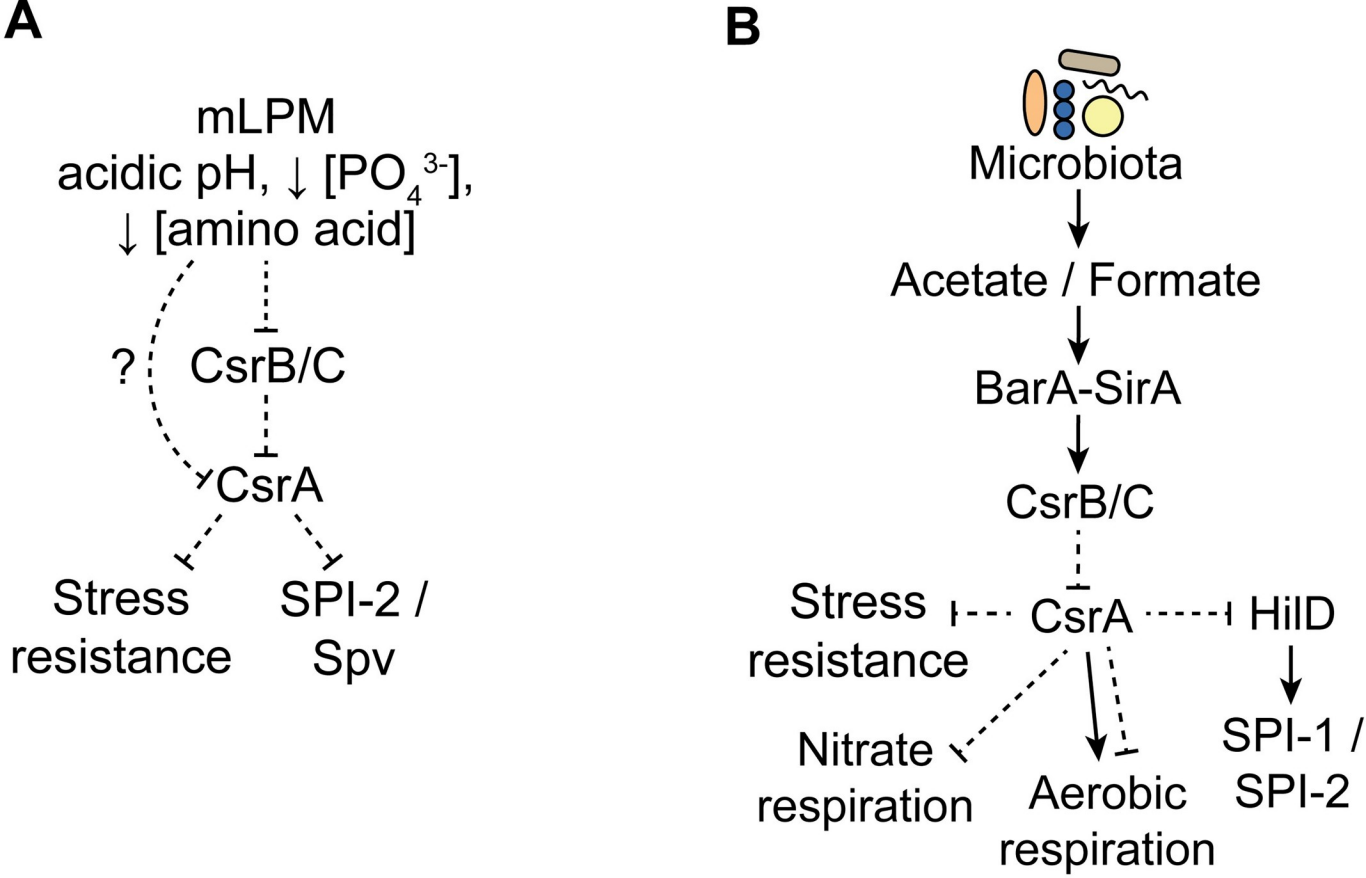
Models of Csr system in mLPM and LB. Dotted lines represent repression and solid lines activation. These arrows represent both direct and indirect effects, as we are unable to differentiate these without further study.

Growth in mLPM minimal medium resulted in decreased CsrB/C levels (Fig 3 and S1 Table). We were able to identify some of the cues that contribute to this difference, including phosphate limitation, acidic pH, and carbon source (Figs 3 and 8A). Studies in *E*. *coli* showed that growth in a nutrient poor medium can result in increased CsrB/C expression, while addition of tryptone or casamino acids caused a decrease in CsrB/C expression [123]. However, our data show that growth of *Salmonella* in a different nutrient poor medium (mLPM) resulted in a strong decrease in CsrB/C levels compared to LB, which supplies amino acids and oligopeptides (S1 Table). Furthermore, the addition of casamino acids to mLPM minimal medium led to an increase in CsrB levels in *Salmonella* (Fig 3). Thus, the conclusion that nutrient poor growth conditions inevitably lead to higher expression of CsrB/C while amino acids cause decreased CsrB/C expression appears to be incorrect or overly simplistic in *Salmonella*. Due to the importance of CsrB/C sRNAs on CsrA activity, identifying additional cues and signals that cause their levels to increase in mLPM in response to the addition of phosphorus, amino acids or increased pH is worthy of additional investigation.

To derive information about genes that are regulated by CsrA during infection, we use findings observed here *in vitro* to develop hypotheses about what may be occurring in the intestine or at systemic sites, in which *Salmonella* is typically located intracellularly. These hypotheses gain some validity from the observation that growth in LB induces the expression of SPI-1, which is important for *Salmonella* to inflame the intestine [31,124], and mLPM induces SPI-2, which is important for *Salmonella* survival at systemic sites and within host cells. We are most likely to observe regulation by CsrA for infection-relevant genes that are highly transcribed *in vitro*, such as SPI-1, SPI-2, and those involved in central metabolism. We are less likely to observe regulation of infection-relevant genes that are poorly transcribed *in vitro*, such as those encoding nutrient acquisition systems, for which the nutrients are not present in the growth media used here.

Generally, the magnitude of CsrA-dependent effects on gene expression in mLPM medium was less pronounced than in LB (S3 Fig and S4 Table). This is in spite of no apparent change in CsrA expression and a pronounced decrease in CsrB/C levels in mLPM (Fig 3 and S1 Table). Together this suggests that there might be other repressors of CsrA activity functioning in mLPM medium. The 5’-UTR of the *fimAICDHF* transcript is known to act as a CsrA titrating factor in *Salmonella* [125]. However, we did not detect significant expression of this transcript in our study (S1 Table). In *E*. *coli*, the two sRNAs McaS and GadY have been proposed to act as CsrA titration factors [126,127]. In addition, recent publications identified a chaperone protein in enteropathogenic *E*. *coli* and several sRNAs in both *E*. *coli* and *Salmonella* that interact with CsrA *in vivo* [48,52,128]. It is unclear if these sRNAs or other CsrA binding factors are responsible for the reduced CsrA activity in mLPM. Despite its seemingly diminished activity in mLPM, CsrA still regulated the expression of many genes involved in stress resistance and virulence (S2 Table, Fig 8A).

The effects of CsrA on gene expression in LB revealed that CsrA regulates a myriad of genes that support competition and survival in the intestine. Consistent with previous work, CsrA repressed expression of *hilD*, which is required for induction of SPI-1 (Fig 8B). There are 17 other potential CsrA binding sites within SPI-1 transcripts, revealed by CLIP-seq, and the majority of the island is strongly repressed by CsrA (S2 Table). Repression of SPI-1 genes was most readily observed in LB due to higher expression but was also observed in mLPM. The T3SS encoded by SPI-1 provokes inflammation of the host, which involves the production of reactive oxygen species that generate oxidized compounds such as tetrathionate and nitrate, which *Salmonella* uses as electron acceptors for respiration and to establish a privileged nutritional niche in a location that is otherwise colonized by native microbiota [57,124,129,130]. CsrA repressed genes involved in anaerobic nitrate respiration and low oxygen aerobic respiration (S2 Table, Fig 8B). CsrA also repressed stress response genes related to its survival in the gut, including osmotic, acid and oxidative stresses (S2 Table, Fig 8). Conceivably, CsrA-mediated repression of *hilD* and other genes required for proliferation in the gut is relieved when *Salmonella* encounters formate and acetate, rich in the intestinal lumen, which activate CsrB/C transcription through the BarA-SirA TCS (Fig 8B).

CsrA also regulated many genes involved in the metabolism of specific substrates in the gut (S2 Table). We caution that these substrates were not added to the media, so we are presumably observing the effects of CsrA on basal level expression. It is plausible that CsrA effects on specific genes might be altered in the presence of the substrates. *Salmonella* can respire compounds in the intestine such as 1,2-propanediol, from the microbiota [131]; ethanolamine, from damaged cells [57]; glucarate and galactarate, from *Nos2*-mediated oxidation of glucose and galactose [132]; L-lactate, from altered host metabolism [133], and fructose-asparagine, from the diet [134–136]. CsrA activated *cbi* genes, for cyanocobalamin synthesis, required for metabolism of ethanolamine and 1,2-propanediol, as well as genes for metabolism of glucarate, galactarate, L-lactate, and fructose-asparagine in LB (S2 Table). In contrast, CsrA repressed *eut* (ethanolamine utilization) genes in mLPM, but not LB. Thus, the Csr system may represent a physiological switch, responsive to intestinal cues, which governs the expression of virulence, metabolic, and stress response genes important for *Salmonella* survival in the gastrointestinal tract.

*Salmonella*-mediated gastroenteritis is typically self-limiting. However, some serovars including Typhi and Paratyphi A, B, or C, can cause a systemic infection of humans called Typhoid or enteric fever. While these serovars cannot infect mice, systemic infection is often modeled using serovar Typhimurium in a mouse model with defective macrophages. In this model, Typhimurium penetrates the intestinal barrier, often using the M-cells of the Peyer’s patches as the portal of entry [137]. The M-cells are destroyed and *Salmonella* is taken up by macrophages where it resides within a vacuole called the SCV, or *Salmonella* containing vacuole [77,138]. This vacuole is acidified, which is required for *Salmonella* to express SPI-2 [80,139]. The T3SS encoded by SPI-2 then secretes effectors that modify host cell trafficking in ways that prevent *Salmonella* destruction and allow growth [12,140]. Transcription profiling of *Salmonella* that is residing within host cells demonstrates that numerous virulence genes are activated in addition to a variety of metabolic pathways and nutrient acquisition systems [141]. It is known that CsrA regulates SPI-2 [31], which is required for intracellular survival, and consistent with this, eight CsrA binding sites within SPI-2 mRNAs were identified by CLIP-seq (S3 Table) [52]. Additionally, CsrA weakly activates SPI-2 gene expression in mLPM and represses their expression in LB (S2 Table).

While the details of the trafficking alterations that are induced by SPI-2 and the nutrient composition of the SCV are under intense investigation, it is clear that *Salmonella* metabolism within these compartments is complex, involving over 31 nutrients that are used with considerable plasticity [142]. Bumann and coworkers used a combination of approaches to model *Salmonella* metabolism in the spleen of infected mice. They observed that *Salmonella* uses a diversity of nutrients in this situation including glycerol, fatty acids, *N*-acetylglucosamine, glucose, lactate, and arginine. Except for glycerol, these nutrients are not present in our minimal mLPM, but we may observe CsrA-dependent regulation of basal levels of transcription. Indeed, *glpF*, encoding the glycerol uptake facilitator protein, was activated by CsrA in mLPM, and there are CsrA binding sites identified by CLIP-seq on both *glpF* and *glpK*, encoding the glycerol kinase (S2 Table). The fatty acid transporter *fadL* has a CsrA binding site identified by CLIP-seq, but we did not observe regulation by CsrA, possibly due to a lack of fatty acids in the media. Similarly, CLIP-seq identified a CsrA binding site on the *nagA* gene encoding N-acetylglucosamine-6-phosphate deacetylase, and on the *artI* gene, encoding a component of an arginine transporter, although their regulation by CsrA was not observed under the conditions used here. It is important to remember that the population of *Salmonella* in the spleen is likely to be heterogeneous with some bacteria being extracellular, and those that are intracellular being present in different intracellular environments. These fine details of *Salmonella* metabolism and its regulation by CsrA await further detailed studies.

## Materials and methods

### Bacterial strains and culture media

The *csrA* mutant strain was constructed from a wild type *Salmonella enterica* subsp. *enterica* serovar Typhimurium str. 14028s parental strain (ATCC). The last 30 base pairs of the *csrA* coding region were replaced with a chloramphenicol resistance marked using the lambda red recombinase method, which was subsequently removed with flippase [143]. This *csrA* mutant replicates the truncated form of CsrA encoded by the *csrA*::*kan* transposon mutant allele used widely in *E*. *coli*, and is genetically stable, unlike *csrA* deletion strains [32,35,60]. In addition, the growth of this *csrA* truncation mutant was the same as its isogenic parental strain under the conditions of this study (S1 Fig). Bacterial cultures were grown at 37°C with shaking at 250 rpm. Cultures were grown overnight in LB medium (1% NaCl, 1% tryptone, and 0.5% yeast extract) then subcultured into LB and grown to mid-exponential phase. These exponentially growing cultures were used as inocula for all experiments. For experiments in LB, cultures were grown from an initial OD_600_ of 0.01 and collected at mid-exponential phase (OD_600_ 0.5). For experiments in mLPM (5 mM KCl, 7.5 mM (NH_4_)_2_SO_4_, 0.5 mM K_2_SO_4_, 0.3% (vol/vol) glycerol, 0.0001% thiamine, 0.5 μM ferric citrate, 8 μM MgCl_2_, 337 μM PO_4_^3-^, and 80 mM MES, pH 5.8)[43], cultures were grown from an initial OD_600_ of 0.1 and collected at mid-exponential phase (OD_600_ 0.3) except for experiments measuring CsrB levels in response to mLPM exposure.

### Ribosome profiling and paired RNA-seq analysis

Ribosome profiling was carried out as previously described [48,144,145]. Briefly, ribosome protected fragments were purified from ribosomes isolated by ultracentrifugation of micrococcal nuclease treated cell lysate through a sucrose cushion. Total RNA was isolated in parallel, depleted of rRNA, and fragmented with alkaline hydrolysis. RPF and RNA fragments of 25–40 bases were then size selected with denaturing PAGE. Sequencing libraries were prepared as described previously using primers in S5 Table [48,144]. Libraries were sequenced by the Genomic Services Laboratory at HudsonAlpha with 50SE Hiseq 2500 (Illumina). Raw sequencing reads were demultiplexed, trimmed, depleted of rRNA sequences, and mapped to the *Salmonella enterica* serovar Typhimurium strain 14028s genome and plasmid sequences (NC_016856.1 and NC_016855.1) with bowtie [146]. Read counts per gene were calculated with htseq-count [147]. Genes with less than an average of 10 counts per gene in all samples were eliminated. Differential expression analysis was carried out with limma voom with quality weights [148]. Analysis of RNA abundance (*RNA_csrA_*–*RNA_WT_*), translation (*RPF_csrA_*–*RPF_WT_*), and translation efficiency ([*RPF_csrA_*–*RPF_WT_*]–[*RNA_csrA_*–*RNA_WT_*]) was combined into a single model of expression for protein coding genes only, allowing us to analyze the contribution of each to the gene’s overall expression. Multiple testing correction was conducted at the gene level using a nested F test, which weighs comparisons with multiple significant contrast more heavily. Comparisons were considered significant when the log_2_ fold change was greater than ±0.8 and the F test was significant. All genes including protein coding and noncoding genes were analyzed together for differences in RNA abundances (*RNA_csrA_*–*RNA_WT_*). These comparisons were considered significant when the log_2_ fold change was greater than ±0.8 and the false discovery rate was less than 0.05.

### RNA stability analysis

RNA stability was analyzed as described previously with some modifications [48]. At the mid-exponential phase, cultures were treated with 500 μg ml^-1^ of rifampicin to stop transcription initiation. Samples were collected after 0, 2.5, 5, 10, and 15 minutes and added directly to a stop solution (5% water saturated phenol in ethanol). RNA was purified with hot phenol chloroform extraction followed by ethanol precipitation. Genomic DNA contamination was removed by treatment with Turbo DNase (ThermoFisher), and RNA was purified with the RNeasy kit (Qiagen). Samples were sent to BGI America for rRNA depletion, sequencing library preparation, and sequencing with 100PE Hiseq 2000 (Illumina). Raw sequencing reads were demultiplexed, trimmed, depleted of rRNA sequences, and mapped to the *Salmonella enterica* serovar Typhimurium strain 14028s genome and plasmid sequences (NC_016856.1 and NC_016855.1) with bowtie2 [149]. Scaling factors were calculated for each sample at each time point by comparing RNA abundance calculated with qRT-PCR to RNA-seq, as described previously [47]. Normalization factors were calculated for each sample using the geometric average of scaling factors calculated from 11 genes (STM14_0169, STM14_0523, STM14_0577, STM14_0882, STM14_1056, STM14_1594, STM14_2331, STM14_2356, STM14_2422, STM14_2491, and STM14_3077). These genes were chosen to reflect a variety of gene lengths, positions in operons, and RNA stabilities. qRT-PCR RNA decay curves were collected from 4 independent biological replicates. RNA stability was modeled with linear regression on a semi-log plot, and RNA half-lives were calculated with the equation $t_{1/2}=-\frac{ln(2)}{k}$, where k is the slope of the linear regression. Genes with initial read counts less than 75 and R^2^ values less than 0.85 were not analyzed further. Genes with significant changes in their average RNA half-life were identified with two-tailed student’s t-test. Effects of CsrA on stability were considered significant if the log_2_ fold change was greater than ±0.8 and the false discovery rate less than 0.05.

### qRT-PCR

Total RNA was stabilized upon collection by addition to the stop solution then purified with hot phenol chloroform extraction and ethanol precipitation. Turbo DNase (ThermoFisher) was used to degrade genomic DNA, and RNA was purified from these reactions using the RNeasy kit (Qiagen). cDNA was synthesized with Superscript IV (ThermoFisher) using random hexamers as primers. Quantitative reverse transcriptase PCR (qRT-PCR) was conducted with the iTaq Universal SYBR Green Supermix (Bio-Rad) using an iQ5 iCycler real time PCR system (Bio-Rad) for detection. qRT-PCR primer sequences are listed in S5 Table. Reactions of 10 μl consisted of 20 ng of cDNA or DNA standard, 300 nM of each primer, and 1x iTaq universal SYBR Green reaction mix. These reactions were incubated for 1 min of initial denaturation at 95°C, and then 45 cycles of 10 sec of denaturation at 95°C and 20 sec of annealing, extension, and imaging at 60°C. Melt curve analysis was used to verify the specificity of amplifications with the parameters: 95°C for 1 min, 55°C for 1 min, and increasing the temperature 0.5°C/10 sec until reaching 95°C. RNA quantities were determined relative to a standard curve of PCR products using iQ5 analysis software (Bio-Rad) and normalized to 16s rRNA levels.

### Measurement of CsrB levels after transfer to mLPM medium

The wild type strain was grown to mid-exponential phase of growth in LB (OD_600_ = 0.5), washed with phosphate buffered saline (PBS), and added to variants of mLPM medium (OD_600_ = 0.3). mLPM was adjusted with NaOH to pH 7.0 (mLPM, pH 7). The glycerol in mLPM was replaced with 0.3% casamino acids (mLPM + casamino acids). mLPM with supplemented with 1000x higher concentration of MgCl_2_ (final concentration 8 mM, mLPM + Mg) or PO_4_^3-^ (final concentration 337 mM, mLPM + PO_4_). Samples were collected at several time points after the transfer and added to a stop solution. RNA was extracted with hot phenol chloroform extraction and ethanol precipitation. Total RNA (0.25 μg) was separated by denaturing PAGE and blotted onto a positively charged nylon membrane (Roche) with a Mini Trans-Blot Cell (Bio-Rad) according to the manufacturer's instructions. Northern blotting and quantification were carried out as previously described [150].

### Gene Ontology (GO) term enrichment analysis

Genes expressed during growth in our conditions were annotated with GO terms using BLAST2GO software [151]. Enriched GO terms were identified with topGO [152] with Fisher’s exact tests. Only terms associated with more than 3 genes were tested for enrichment. Lists of significant terms were shortened with REViGO [153] to aid visualization. The full lists of enriched terms are presented in S6 Table.

### Oxidative stress assay

Wild type and *csrA* mutant strains were grown in the appropriate media to mid-exponential phase. Hydrogen peroxide was added at the indicated concentrations, and samples were collected before and 0.5, 1, and 2 hours after H_2_O_2_ addition. Samples were washed three times with PBS, serially diluted, and spotted onto LB media. Plates were incubated for 18 hours at 37°C before imaging.

## Supporting information

S1 Table Effect of media on *Salmonella* gene expression. Differential gene expression analysis comparing the effect of growth media (mLPM versus LB) separately for each strain (wild type and *csrA* mutant). There is a key to the right of the table.
(XLSX)

S2 Table Effect of *csrA* mutation on gene expression in mLPM and LB. Differential gene expression analysis comparing the effect of the *csrA* mutation separately for each growth media (mLPM and LB). There is a key to the right of the table.
(XLSX)

S3 Table CLIP-seq peaks in 14028s genome from Holqvist et al. (XLSX)

S4 Table Effects of both media and mutation of *csrA* on gene expression. Differential gene expression analysis testing the interaction between the *csrA* mutation and growth media in a single test. As this analysis is testing the significance of an interaction term, care should be taken in interpreting the biological meaning of a significant result in this comparison. There is a key to the right of the table.
(XLSX)

S5 Table Oligonucleotide sequences. (XLSX)

S6 Table GO term enrichment. There is a key to the right of the table.
(XLSX)

S1 Fig Growth of wild type and *csrA* mutant strains in LB and mLPM. Strains growing exponentially in LB media were subcultured into (A) LB or (B) mLPM media, and growth was measured by optical density at 600 nm.
(TIF)

S2 Fig Ribosome profiling and RNA-seq methods are reproducible. Multidimensional scaling (MDS) plot of (A) ribosome profiling and RNA-seq normalized counts and (B) RNA half-lives.
(TIF)

S3 Fig The effects of CsrA on gene expression in *Salmonella*. Volcano plots showing log_2_ transformed fold change versus log odds comparing wild type and *csrA* mutant strains for translation in (A) mLPM and (B) LB, RNA abundance of protein coding genes in (C) mLPM and (D) LB, translation efficiency in (E) mLPM and (F) LB, and RNA abundance of protein coding and non-protein coding genes in (G) mLPM and (H) LB. Significant comparisons are shown in black.
(TIF)

S4 Fig The effects of CsrA on RNA stability in *Salmonella*. (A) Plot of mean RNA half-life in wild type and *csrA* mutant strains in LB and mLPM. The distributions are significantly difference (Wilcox rank sum test, p < 0.0005: ***). Half-lives of individual genes in (B) mLPM and (C) LB with significant differences shown in black.
(TIF)

S5 Fig Condition specific effects of CsrA on gene expression. Plots showing log_2_ transformed fold change between wild type and *csrA* mutant strains in mLPM versus LB for (A) translation, (B) RNA abundance of protein coding genes, (C) translation efficiency, and (D) RNA stability.
(TIF)

S6 Fig Exposure to mLPM medium reduces CsrB levels. Wild type *Salmonella* was grown to mid-exponential phase in LB media, collected and washed in phosphate buffered saline, and added to mLPM medium. Samples were collected at various time points and RNA immediately stabilized in a phenol ethanol stop solution. (A) 16S rRNA normalized quantification of CsrB levels derived from (B) northern blot data. These data are an independent experimental replication of the data shown in Fig 3.
(TIF)

S7 Fig Extended data for Fig 7B—Survival of wild type and *csrA* mutant strains upon exposure to H_2_O_2_. Strains in mid-exponential phase of growth in mLPM or LB were exposed to 2.5, 5, 10 or 20 μM of H_2_O_2_ for 0, 30, 60, and 120 minutes. Washed and 10-fold serially diluted samples were plated and grown overnight on LB agar before imaging.
(TIF)
