# Supplementary figures and images for: Role of CsrA in stress responses and metabolism important for Salmonella virulence revealed by integrated transcriptomics
Source: PLoS One. 2019 Jan 25;14(1):e0211430. doi: 10.1371/journal.pone.0211430 (PMC6347204; doi:10.1371/journal.pone.0211430)

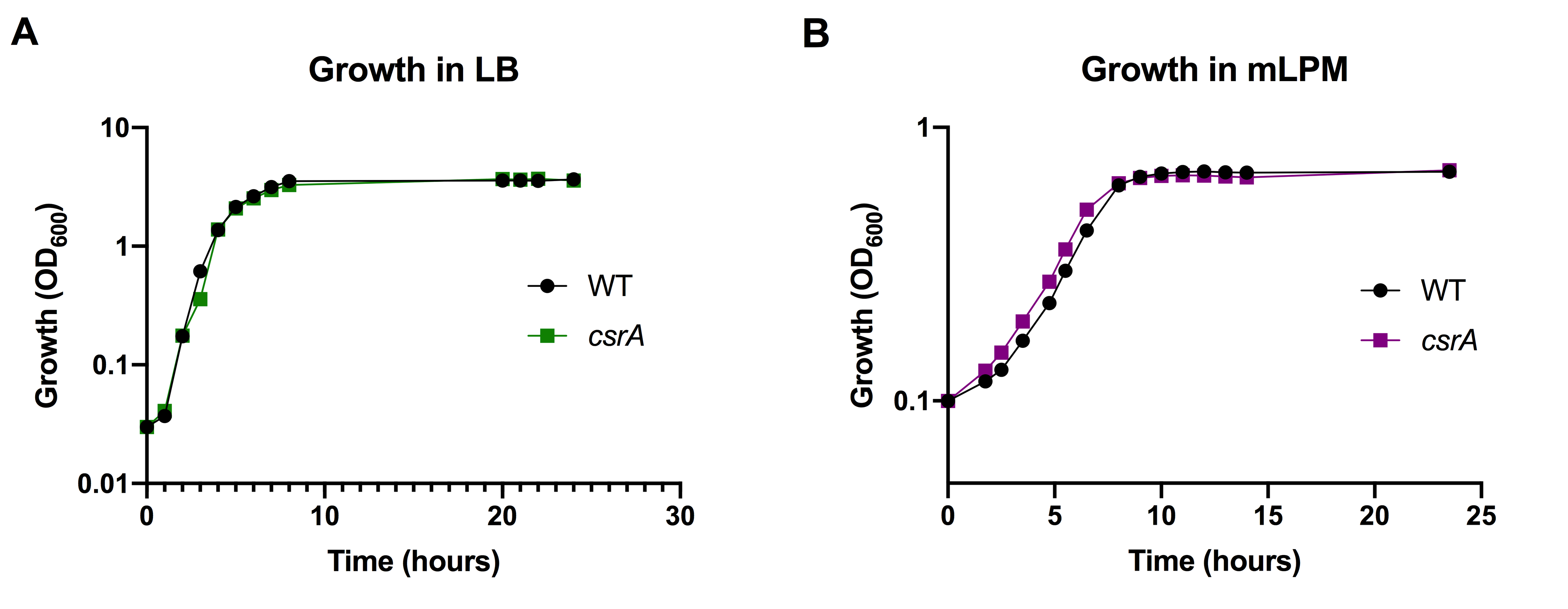

Supplement: S1 Fig — Strains growing exponentially in LB media were subcultured into (A) LB or (B) mLPM media, and growth was measured by optical density at 600 nm. (TIF) [file pone.0211430.s007.tif]

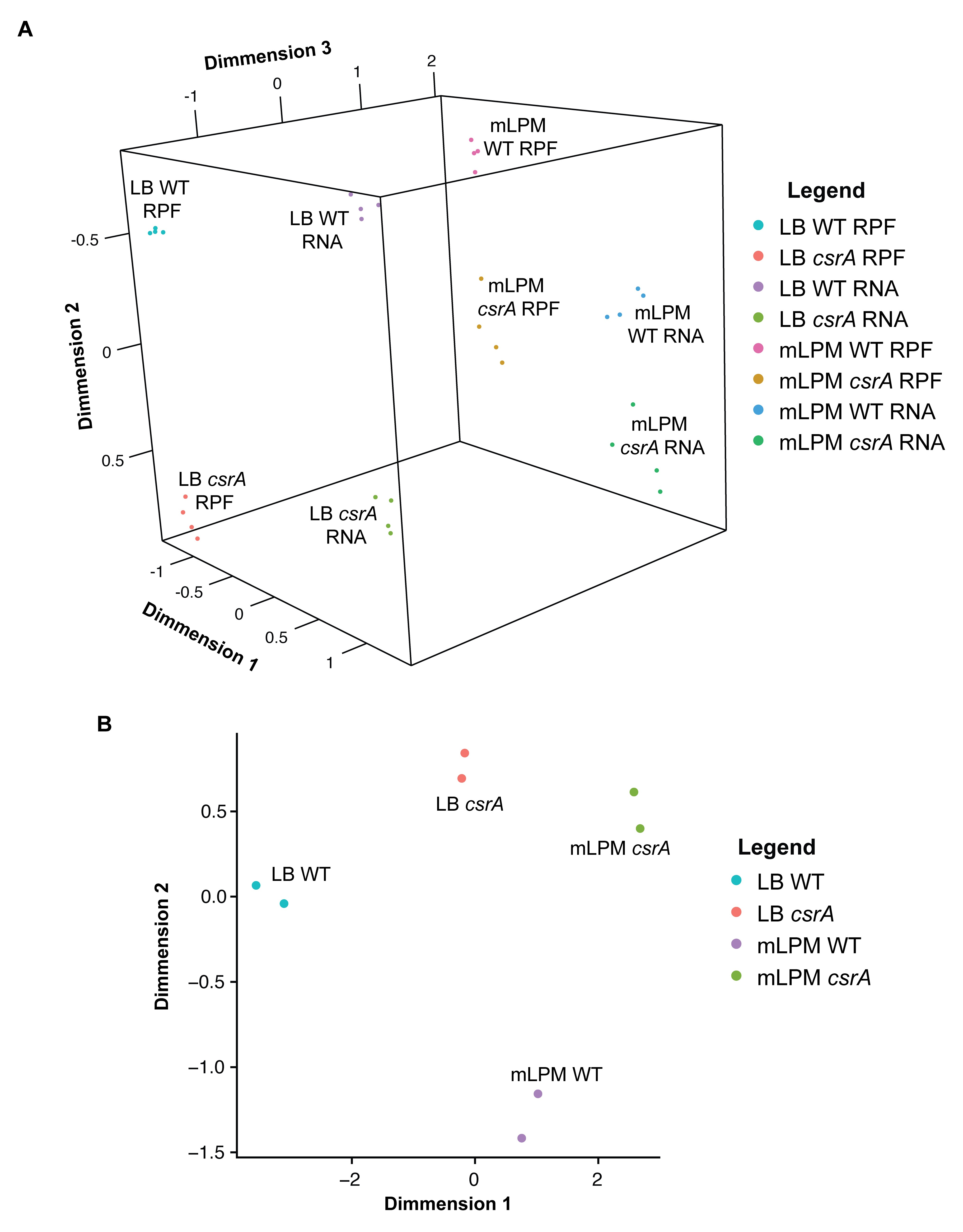

Supplement: S2 Fig — Multidimensional scaling (MDS) plot of (A) ribosome profiling and RNA-seq normalized counts and (B) RNA half-lives. (TIF) [file pone.0211430.s008.tif]

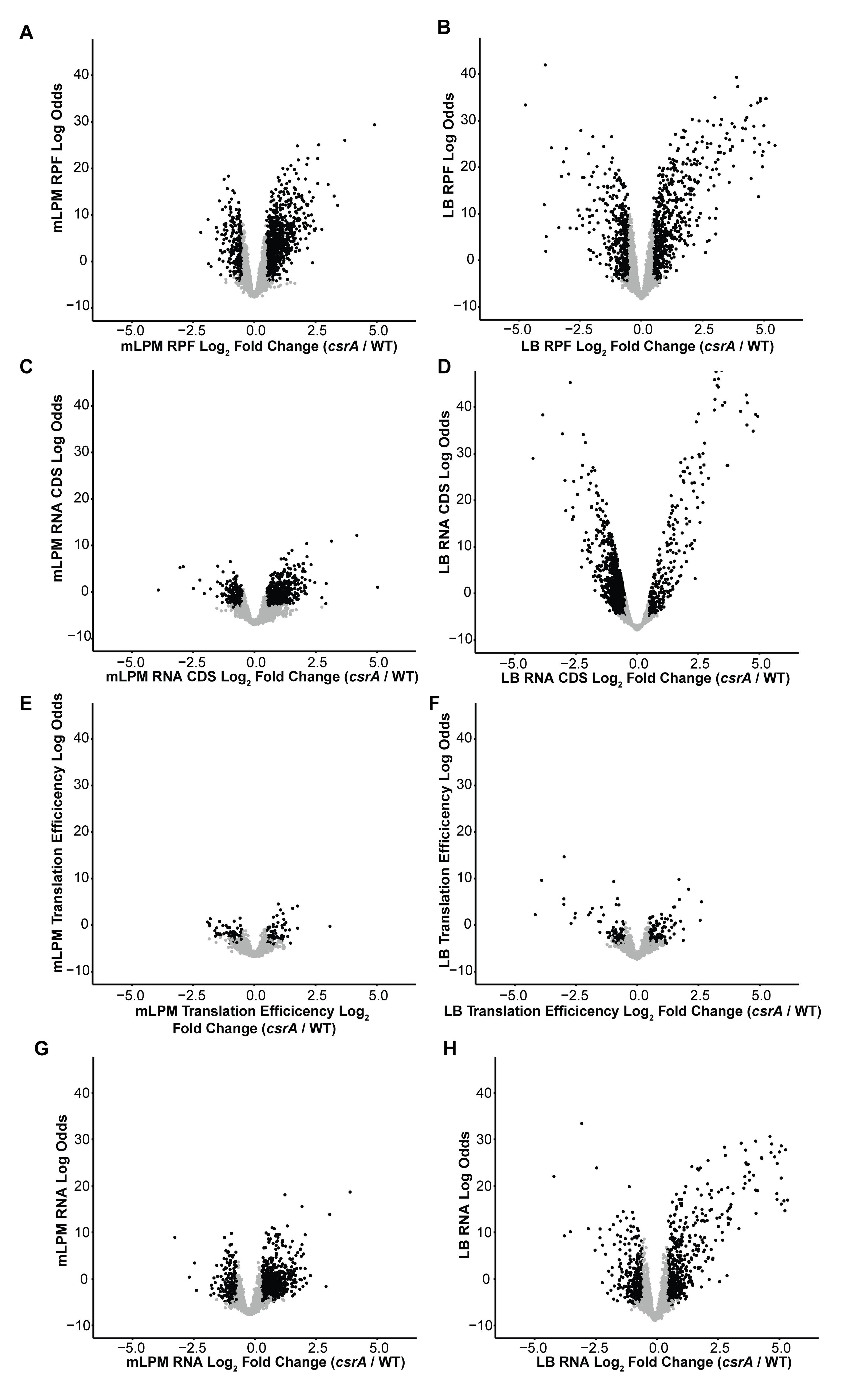

Supplement: S3 Fig — Volcano plots showing log2 transformed fold change versus log odds comparing wild type and csrA mutant strains for translation in (A) mLPM and (B) LB, RNA abundance of protein coding genes in (C) mLPM and (D) LB, translation efficiency in (E) mLPM and (F) LB, and RNA abundance of protein coding and non-protein coding genes in (G) mLPM and (H) LB. Significant comparisons are shown in black. (TIF) [file pone.0211430.s009.tif]

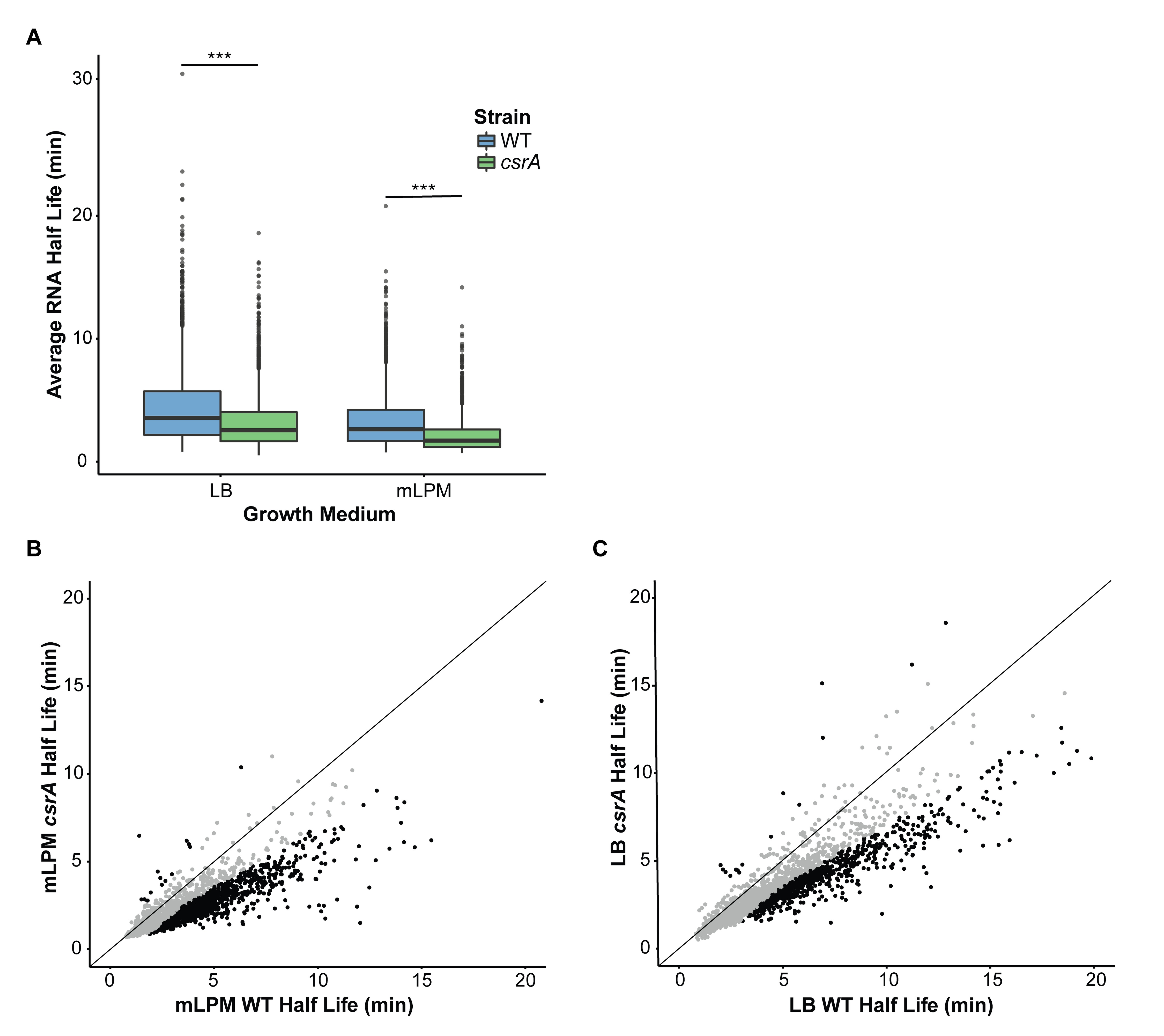

Supplement: S4 Fig — (A) Plot of mean RNA half-life in wild type and csrA mutant strains in LB and mLPM. The distributions are significantly difference (Wilcox rank sum test, p < 0.0005: ***). Half-lives of individual genes in (B) mLPM and (C) LB with significant differences shown in black. (TIF) [file pone.0211430.s010.tif]

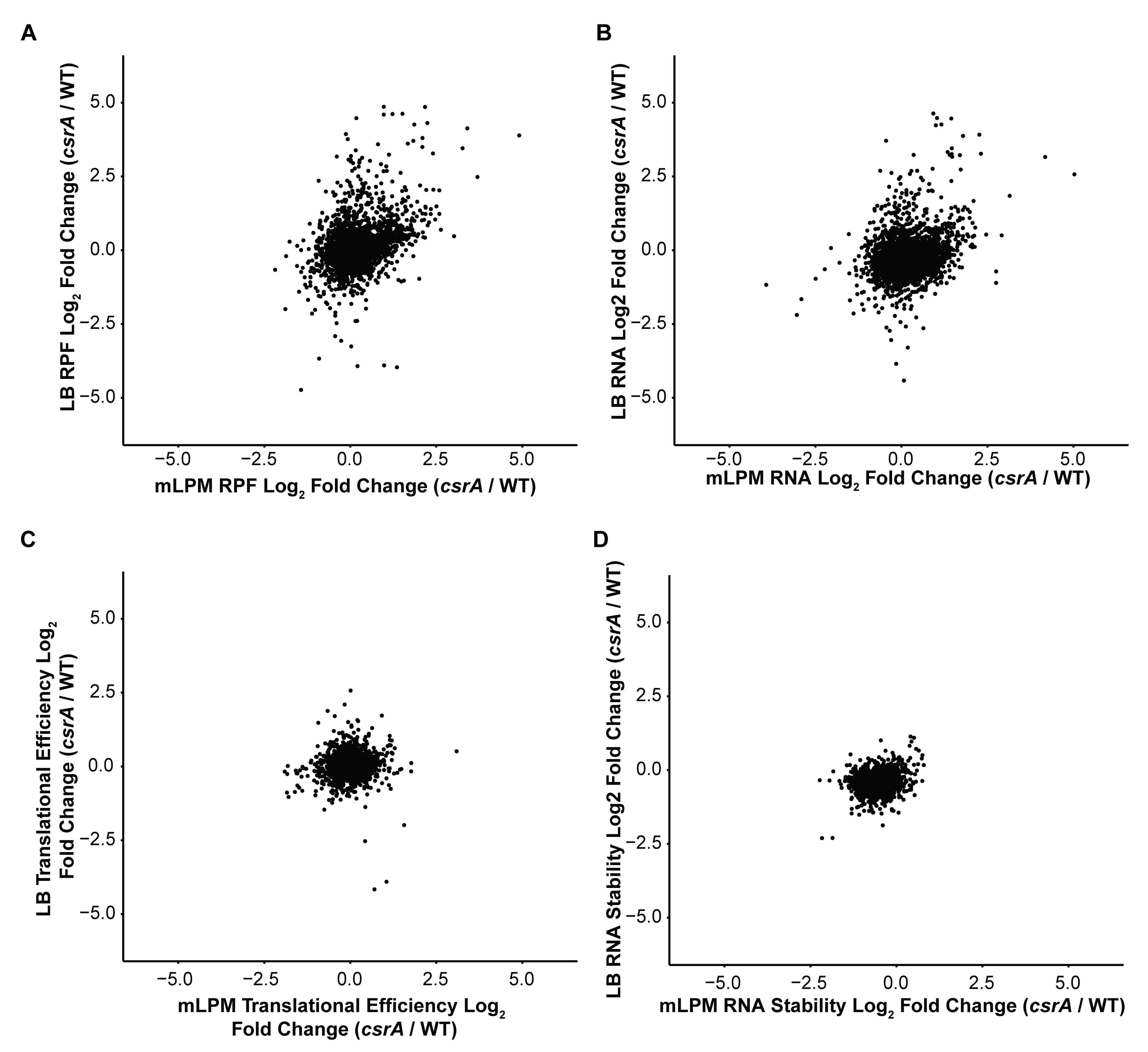

Supplement: S5 Fig — Plots showing log2 transformed fold change between wild type and csrA mutant strains in mLPM versus LB for (A) translation, (B) RNA abundance of protein coding genes, (C) translation efficiency, and (D) RNA stability. (TIF) [file pone.0211430.s011.tif]

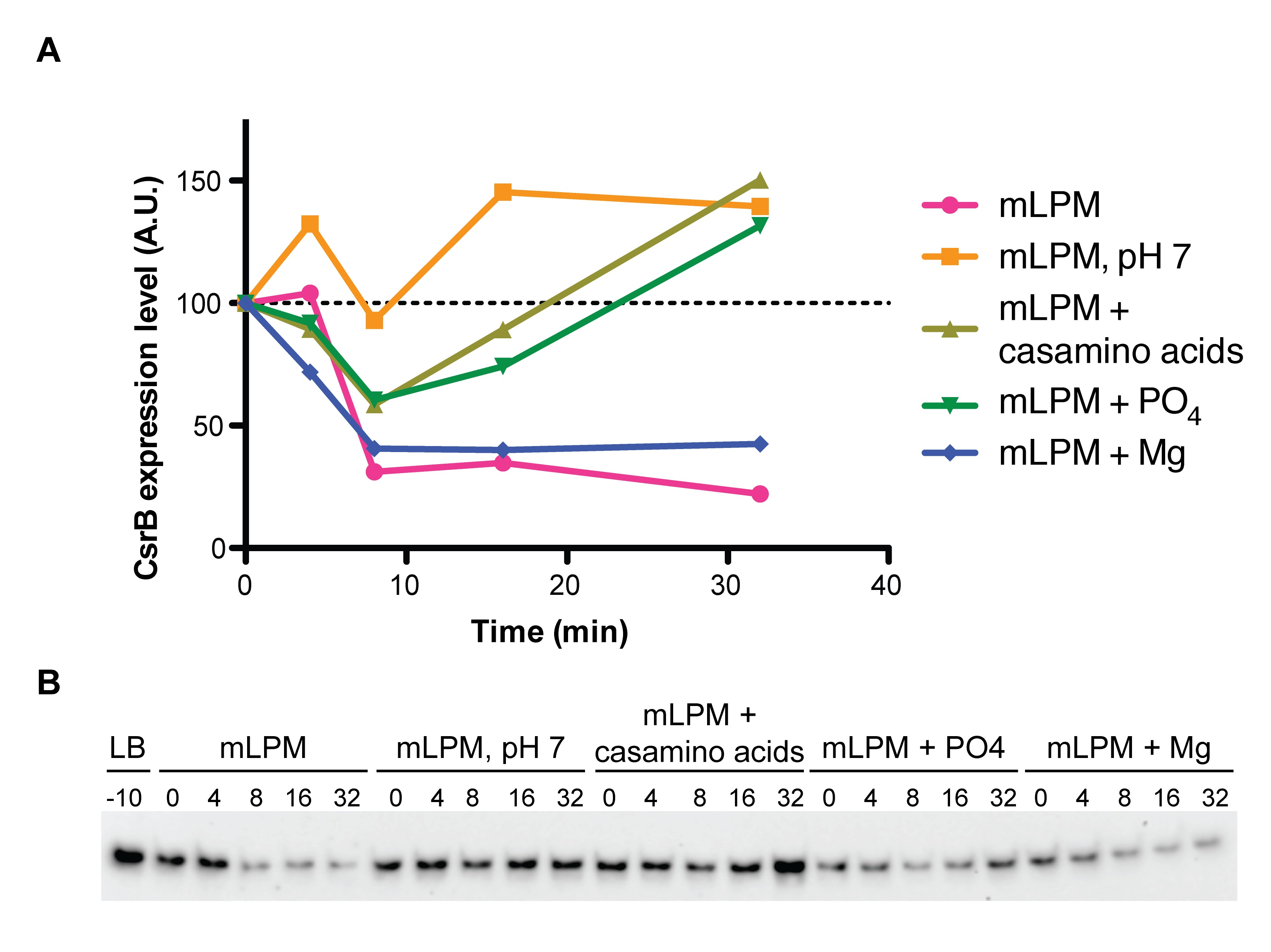

Supplement: S6 Fig — Wild type Salmonella was grown to mid-exponential phase in LB media, collected and washed in phosphate buffered saline, and added to mLPM medium. Samples were collected at various time points and RNA immediately stabilized in a phenol ethanol stop solution. (A) 16S rRNA normalized quantification of CsrB levels derived from (B) northern blot data. These data are an independent experimental replication of the data shown in Fig 3. (TIF) [file pone.0211430.s012.tif]

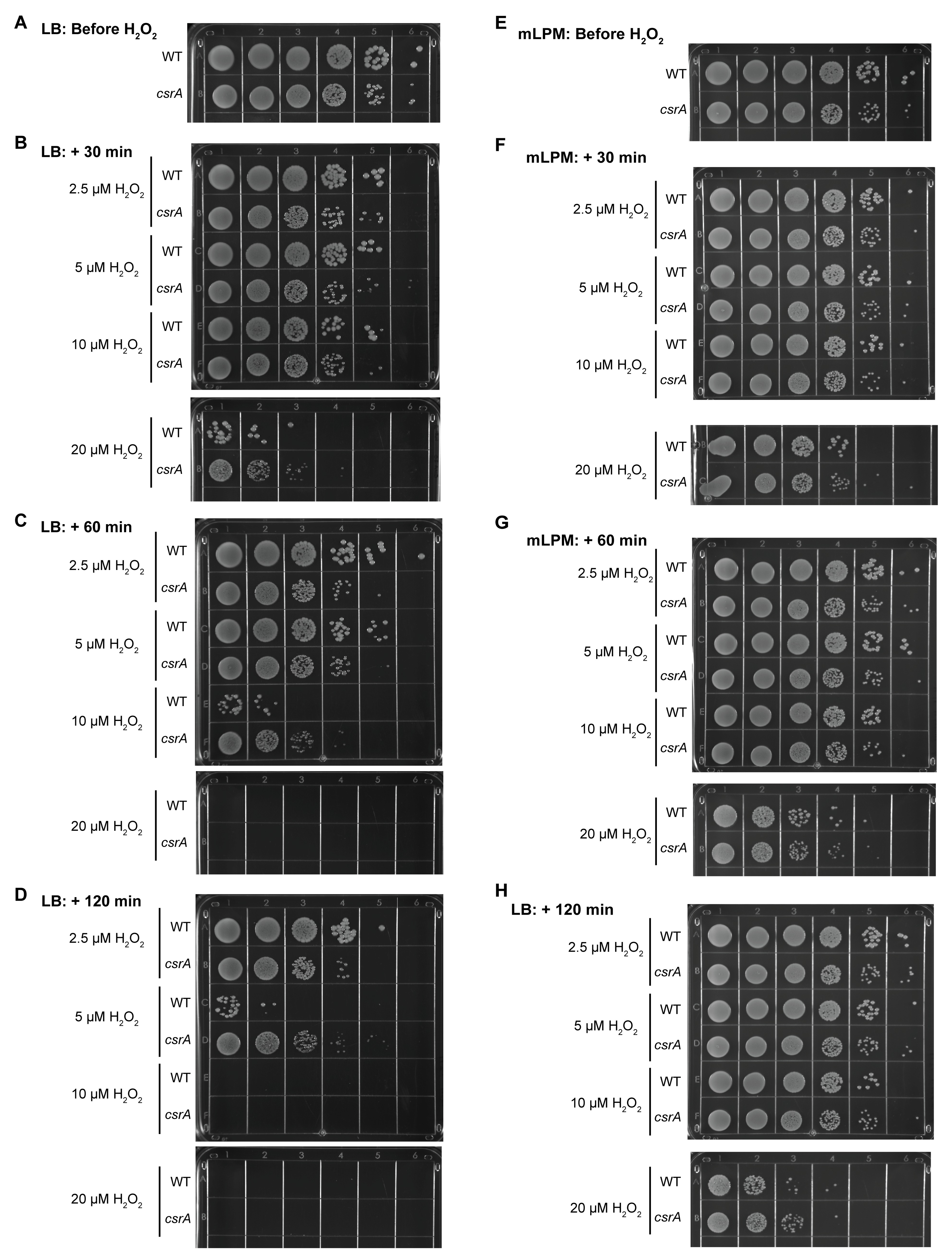

Supplement: S7 Fig — Strains in mid-exponential phase of growth in mLPM or LB were exposed to 2.5, 5, 10 or 20 μM of H2O2 for 0, 30, 60, and 120 minutes. Washed and 10-fold serially diluted samples were plated and grown overnight on LB agar before imaging. (TIF) [file pone.0211430.s013.tif]
